# Pharmacokinetic Properties of ^68^Ga-labelled Folic Acid Conjugates: Improvement Using HEHE Tag

**DOI:** 10.3390/molecules25112712

**Published:** 2020-06-11

**Authors:** Anton Larenkov, Marat Rakhimov, Kristina Lunyova, Olga Klementyeva, Alesya Maruk, Aleksei Machulkin

**Affiliations:** 1 State Research Center–Burnasyan Federal Medical Biophysical Center of Federal Medical Biological Agency, Zhivopisnaya str., bld. 46, 123098 Moscow, Russia; marat.rakhimov89@gmail.com (M.R.); christfmbc@gmail.com (K.L.); klementyeva.olga@gmail.com (O.K.); amaruk@list.ru (A.M.); 2 Department of Chemistry, Lomonosov Moscow State University, GSP-1, Leninskie Gory, 119991 Moscow, Russia; alekseymachulkin@rambler.ru; 3 Department of Semiconductor Electronics & the Physics of Semiconductors, National University of Science and Technology MISiS, 9 Leninskiy pr., 119049 Moscow, Russia

**Keywords:** gallium-68, folic acid, folate receptor, KB cells, histidine-glutamic acid tag, HEHE, pharmacokinetics, kidney accumulation, PET imaging, NODAGA-folate conjugate, radiopharmaceuticals

## Abstract

The folate receptor (FR) is a promising cell membrane-associated target for molecular imaging and radionuclide therapy of cancer (FR-α) and potentially also inflammatory diseases (FR-β) through use of folic acid-based radioconjugate. FR is often overexpressed by cells of epithelial tumors, including tumors of ovary, cervix, endometrium, lungs, kidneys, etc. In healthy tissues, FR can be found in small numbers by the epithelial cells, mainly in the kidneys. Extremely high undesired accumulation of the folate radioconjugates in the renal tissue is a main drawback of FR-targeting concept. In the course of this work, we aimed to reduce the undesirable accumulation of folate radioconjugates in the kidneys by introducing a histidine/glutamic acid tag into their structure. Two folic acid based compounds were synthesized: NODAGA-1,4-butanediamine-folic acid (FA-I, as control) and NODAGA-[Lys-(HE)_2_]-folic acid (FA-II) which contains a (His-Glu)_2_ fragment. In vitro studies with FR (+) cells (KB and others) showed that both compounds have specificity for FR. Introduction of (HE)_2_-tag does not affect FR binding ability of the conjugates. In vivo biodistribution studies with normal laboratory animals, as well as with KB tumor bearing animals, were carried out. The results showed that introduction of the (HE)_2_ tag into the structure of folate radioconjugates can significantly reduce the accumulation of these compounds in non-target tissues and important organs (the accumulation in the kidneys is reduced 2–4 times), leaving the accumulation in tumor at least at the same level, and even increasing it.

## 1. Introduction

Receptor-targeted molecular imaging and therapy are widely recognized as promising tools in oncology [1,2]. Folate receptors (FRs) are expressed at high levels in numerous cancers to meet the folate demand of rapidly dividing cells, which makes these receptors a promising cell membrane-associated target for a number of visualization and treatment techniques including PET imaging of cancer. FRα is the most extensively studied one in a family of high-affinity FRs [3,4,5]. This protein is overexpressed on a variety of tumor types (including ovarian, lung, breast, etc.), while showing low to negligible levels of expression in most normal human tissues. The highest levels of FRα is reported for lungs and kidneys [6,7]. FRβ is another target for molecular imaging since it is overexpressed in macrophages [8,9].

High kidney uptake of FR-targeted preparations is caused by FRα expression in proximal tubules. Regarding nuclear medicine applications for the moment high kidney uptake is considered to be the most challenging obstacle in the development of folate-based radiopharmaceuticals [5].

Several folic acid radioconjugates have been proposed as promising for imaging and therapy of FRα-positive cancers [10]. Only few of them have been studied in human patients [11,12,13,14,15]. In the ongoing clinical trial the first ^68^Ga-folate PET radiotracer ([^68^Ga]Ga-EC2115) is tested to evaluate the possibility of differentiating COPD (chronic obstructive pulmonary disease) patients from control subjects and determine whether PET signal correlates with measurements of inflammation, disease severity, and rate of disease progression [16]. Currently, data on several folic acid based radiopharmaceuticals with PET-radionuclides, such as ^18^F, ^68/66^Ga, ^152^Tb, ^64^Cu, ^55^Co and ^44^Sc have been presented and even more new conjugates are under development [17,18,19,20,21,22,23].

The concept of using folic acid as a targeting agent is undoubtedly very promising on account of it being a small molecule (441 Da), which is accessible for chemical modification in order to bind virtually any diagnostic or therapeutic isotope without losing binding affinity to FR [24]. The reason why folate radioconjugates have been still scarcely used in clinics so far may be related to their above-mentioned undesired accumulation in the renal tissue. An admirable approach was proposed by Müller et al. to fix this drawback of the FR-targeting concept [25]. By introducing an albumin-binding fragment into DOTA-conjugated folic acid the prolong blood circulation time was achieved which resulted in an increased tumor uptake and reduced retention of radioactivity in the kidneys. This modification allowed to carry out first FR-targeted radionuclide therapy study in mice. Survival median in ^177^Lu-treated groups of animals significantly expanded in comparison to the control group. Later this approach was used with ^64^Cu- and ^68^Ga labeled NODAGA-folate derivates [17].

Besides high tumor uptake, fast background clearance of radiolabeled molecules from blood and critical organs typically correlates with higher imaging contrasts and consequently with higher probability to influence the subsequent therapy strategy. Thus, any approach optimizing the pharmacokinetic characteristics of radiopharmaceuticals results in better imaging contrast. An interesting approach was introduced by Tolmachev’s group from Uppsala University. In [26,27] the advantageous effect of introducing a histidine/glutamic acid tag (HEHEHE-tag or (HE)_3_-tag) on the biodistribution of anti-HER2 Affibody molecules was reported. The modification resulted in a reduced liver uptake while the high affinity of the affibody has been maintained. Later it was shown that although in some cases this modification of affibodies may lead to lower tumor uptake, still (HE)_3_-tagged variants are favorable to non-tagged variants for in vivo imaging. Consequently, modified anti-HER2 affibodies [^68^Ga]Ga-(HE)_3_-Z_08698_-NOTA and [^68^Ga]Ga-(HE)_3_-Z_08698_-NODAGA demonstrated superior tumor-to-liver ratios compared with [^68^Ga]Ga-Z_08698_-NOTA and [^68^Ga]Ga-Z_08698_-NODAGA [28]. The study of Eder et al. has shown that introducing (HE)_3_-tag into another vector molecule (PSMA-11) leads to considerable drop in kidney and liver uptake as well as in blood retention. Notably, in this case tumor uptake was as high as that for the original [^68^Ga]Ga-PSMA-11 [29,30,31]. This effect optimizes imaging contrast and can potentially lead to a better management of metastatic disease. In a number of cases tumor uptake of tagged radiotracers was higher than that for original molecules [30]. These data are very impressive and promising in terms of further modification of various radiopharmaceuticals. We have not found any published work on the influence of (HE)_n_-tag modification of the pharmacokinetics of radiolabeled folic acid conjugates.

**Figure 1:**
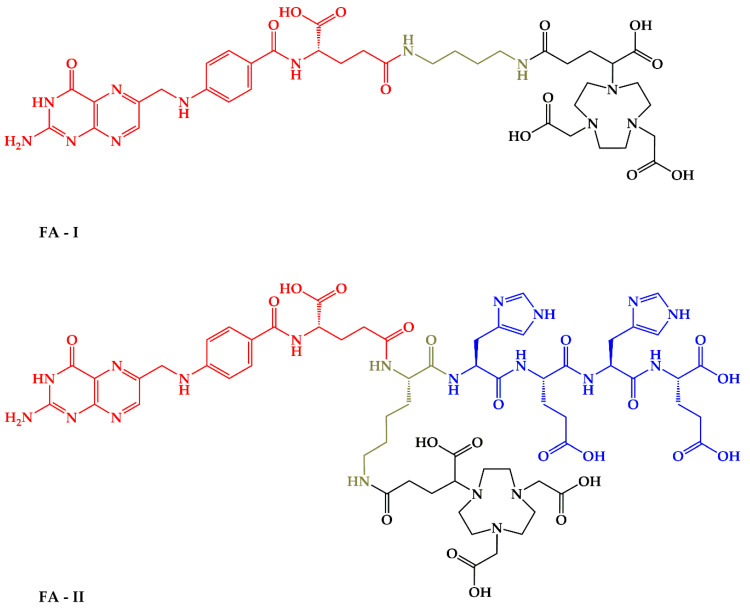
Chemical structure of **FA-I** and **FA-II** with marked moieties: red-folic acid vector moiety; black–NODAGA chelator for ^68^Ga coordination; green–linkers; blue–(HE)_2_-tag for pharmacokinetic properties optimization.

Thus, the aim of this work is the evaluation of the effect of (HE)_n_-tag on the biodistribution profile of the low-molecular weight receptorspecific NODAGA-folic acid bioconjugate labeled with gallium-68. For this purpose, two molecules were synthesized: NODAGA-1,4-butanediamine-folic acid (**FA-I**) and NODAGA-[Lys-(HE)_2_]-folic acid (**FA-II**)–Figure 1. The first molecule (**FA-I**) is the closest analogue of folate-based radiopharmaceuticals with NODAGA chelator reported in the literature [32]. The second one (**FA-II**) is the new molecule containing (HE)_2_-tag in its structure. After labeling with ^68^Ga both molecules have been tested in vitro and in vivo.

## 2. Results

### 2.1. Synthesis of Precursors

**Scheme 1:**
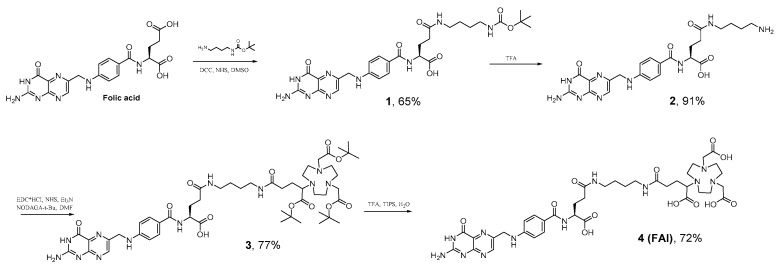
Synthesis NODAGA-1,4-buthanediamine--folic acid (**FA-I**).

NODAGA-1,4-butanediamine-folic acid (**FA-I**) was synthesized according to Scheme 1. Briefly, modification of folic acid with *N*-Boc-1,4-butanediamine was performed according to a simple two step liquid phase protocol previously described by Trindade et. al. for *N*-Boc-1,2-ethylenediamine [33]. 

Compound **1** was obtained in 65% yield. Subsequent deprotection was carried out in TFA solution, followed by further precipitation with TEA and washing. These procedures gave a yellow powder of **2**, which was further modified with NODAGA(tBu)_3_ followed by deprotection of *tert*-butyl esters with TFA/TIPS/H_2_O (95/2.5/2.5) solution resulting in **4** (**FA-I**). The overall yield of the synthesis carried out according to Scheme 1 is 33%. The purity of final **FA-I** product is 91% (Figure S12).

**Scheme 2:**
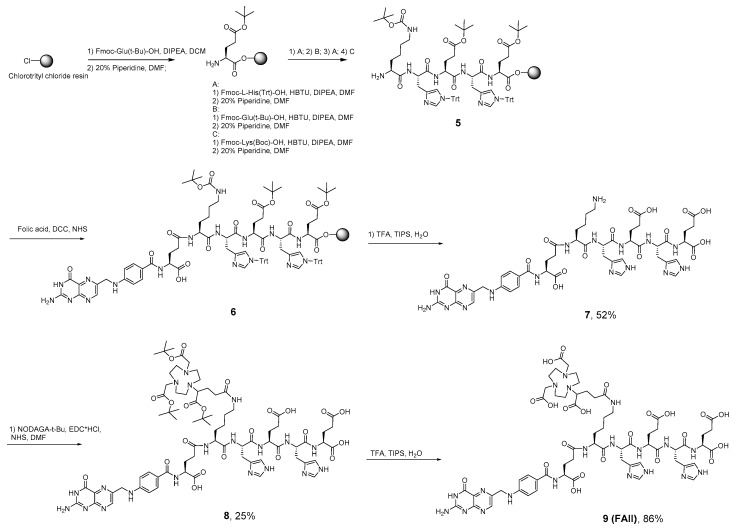
Synthesis NODAGA-[Lys-(HE)_2_]--folic acid (**FA-II**).

NODAGA-[Lys-(HE)_2_]-folic acid (**FA-II**) was synthesized according to Scheme 2. The trityl- and *tert*-butyl and Boc-protected KHEHE sequence was synthesized according to standard Fmoc protocols using 2-chlorotrityl chloride resin and the respective trityl- and *tert*-butyl–protected amino acids [29]. Modification of KHEHE sequence with folic acid was performed on a resin with PyBOP to generate an active ester in situ and grafted onto the resin via formation of an amide bond as it was described previously by Atkinson et al. [34]. Subsequent step consisted of the cleavage of the functionalized folic acid from the resin under acidic conditions of TFA/TIPS/H_2_O (95/2.5/2.5), resulted in compound **7**, obtained as an orange powder. Next, compound **7** was modified with NODAGA(tBu)_3_ with final deprotection of *tert*-butyl esters with TFA/TIPS/H_2_O mixture, which resulted in yellow powder of desired compound **9** (**FA-II**). The overall yield of the synthesis carried out according to Scheme 2 is 11%. The purity of final **FA-II** product is ≥ 99% (Figure S13).

### 2.2. Radiolabelling, Stabillity and Distribution Coefficients (LogD_ow_ value) Studies

Due to the use of NODAGA bifunctional chelating agent in the structure of the synthesized molecules, high yield (≥ 98%) of the radiolabeling was achieved (the content of hydrolyzed (colloidal) gallium-68 is ≤ 1%; the content of unbound ionic gallium-68 ≤ 1%) for both compounds after 10 min of incubation of the reaction mixture (12–20 μg of precursors, 0.3 M sodium acetate and 150 MBq ^68^Ga in 1 mL, pH 4.5) at room temperature (RT). 

Both [^68^Ga]**Ga-FA-I** and [^68^Ga]**Ga-FA-II** demonstrated high stability in saline, PBS, and human apo-transferrin solution for at least 2 h. The plasma stability of [^68^Ga]**Ga-FA-I** and [^68^Ga]**Ga-FA-II** was determined in human plasma in vitro. HPLC and TLC analysis of the samples revealed that both radiotracers remained intact ( > 99%) during incubation at 37 °C for at least 3 h.

Log*D*_ow_ value were determined to obtain information about the hydrophilicity of the radiolabeled folate conjugates. For [^68^Ga]**Ga-FA-I** a log*D*_ow_ value of −2.65 ± 0.34 and for [^68^Ga]**Ga-FA-II** a log*D*_ow_ value of −2.86 ± 0.41 were obtained. There was no significant difference between these values (*p* > 0.05).

### 2.3. In Vitro Studies

**Figure 2:**
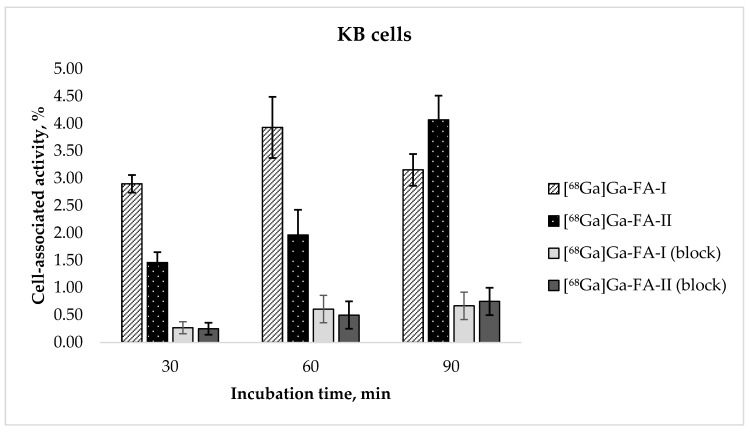
In vitro accumulation of [^68^Ga]**Ga-FA-I** and [^68^Ga]**Ga-FA-II** in KB cell culture (*mean ± SD, n = 3*).

The results of the evaluation of receptor specific cell binding ability [^68^Ga]**Ga-FA-I** and [^68^Ga]**Ga-FA-II** obtained with FR(+) KB cells are presented in Figure 2. After 30 and 60 min of incubation cell-associated activity of [^68^Ga]**Ga-FA-II** was nearly twice lower than that of [^68^Ga]**Ga-FA-I** (e.g., 3.93 ± 0.56% for [^68^Ga]**Ga-FA-I** vs. 1.97 ± 0.46% for [^68^Ga]**Ga-FA-II** after 60 min of incubation, *p* < 0.01). Continued exposure of the cells to the radiofolates resulted in cell uptake of [^68^Ga]**Ga-FA-II** after 90 min (4.07 ± 0.44%) virtually equal to that of [^68^Ga]**Ga-FA-I** after 60 min (*p* > 0.05). For [^68^Ga]**Ga-FA-I** no further increase of cell uptake was observed (3.16 ± 0.29%, *p* > 0.05). It is probable that introduction of (HE)_2_-tag leads to some sort of steric constraint resulting in slower dynamics of molecule-receptor interaction. Blocking experiments performed with presaturation of cell culture with 100-fold excess of native folic acid showed low nonspecific binding of [^68^Ga]**Ga-FA-II**. However [^68^Ga]**Ga-FA-I** accumulation in KB cells is significantly lower than that reported for closest analog in the literature (see Discussion section).

**Figure 3:**
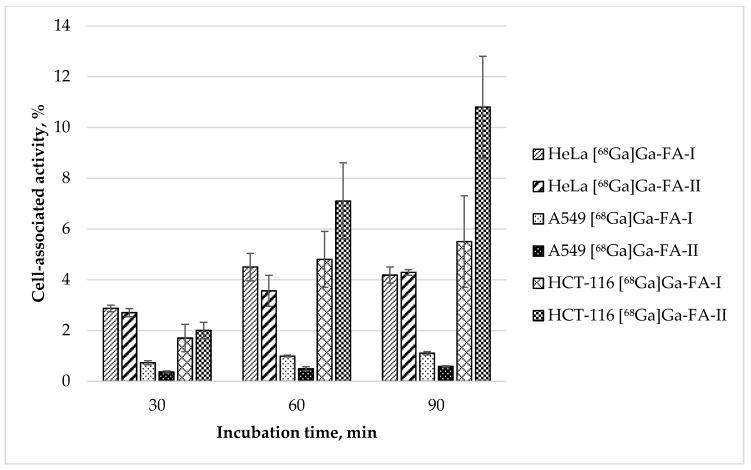
In vitro accumulation of [^68^Ga]**Ga-FA-I** and [^68^Ga]**Ga-FA-II** in HeLa, A549 and HCT-116 cell cultures (*mean ± SD, n = 3*).

Alongside with KB cells widely used for folate-based radiopharmaceuticals in vitro accumulation studies, [^68^Ga]**Ga-FA-I** and [^68^Ga]**Ga-FA-II** uptake was also evaluated for HeLa (FR(+), as progenitor of KB cells [35]), A549 (FR(−); as control) and HCT-116 (FR(+) [36,37]) cells. The results of these in vitro tests are presented in Figure 3.

Experimental data show that the uptake of both labelled compounds in HeLa cell line is virtually equal: 4.05 ± 0.54 and 3.56 ± 0.61% after 60 min, and 4.18 ± 0.32 and 4.29 ± 0.11% after 90 min for [^68^Ga]**Ga-FA-I** and [^68^Ga]**Ga-FA-II** correspondingly (*p* > 0.05). These data are similar to those obtained with KB cells. In contrast to that, accumulation of both labelled compounds in FR(−) A549 cells was exceptionally low (1.11 ± 0.07 and 0.59 ± 0.03% after 90 min for [^68^Ga]**Ga-FA-I** and [^68^Ga]**Ga-FA-II** correspondingly). A549 cells uptake is comparable to nonspecific uptake obtained with folic acid blocked KB cells. Surprising is the fact that HCT-116 cells showed [^68^Ga]**Ga-FA-II** accumulation not only higher than that for HeLa and BK cells, but also higher than [^68^Ga]**Ga-FA-I** accumulation in same HCT-116 cell culture: 10.8 ± 1.9% vs. 5.5 ± 1.7% after 90 min of incubation (*p* < 0.05). This fact is an interesting object for future investigation, however, since KB cells are standard for folate-based radiopharmaceuticals evaluation, under the scope of this study in further in vivo experiments KB xenografts were used. 

### 2.4. In Vivo Studies

#### 2.4.1. Biodistribution in Healthy Animals

**Figure 4:**
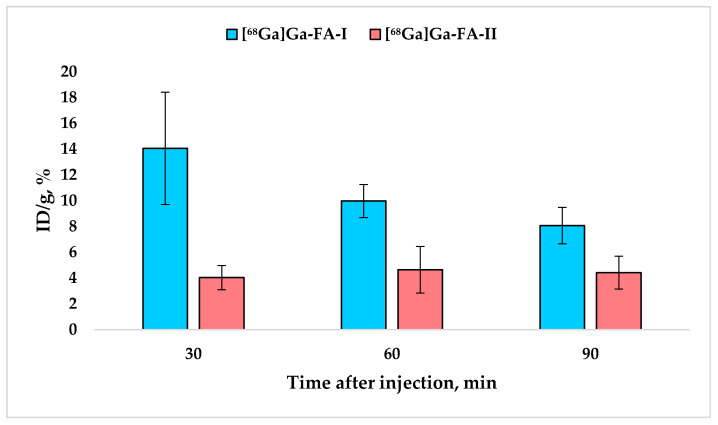
Accumulation of [^68^Ga]**Ga-FA-I** and [^68^Ga]**Ga-FA-II** in kidneys (*mean ± SD, n = 5*).

Biodistribution results obtained in Wistar rats for [^68^Ga]**Ga-FA-I** and [^68^Ga]**Ga-FA-II** are presented in Table 1. The results show that in normal animals labeled compounds under study are mainly accumulated in kidneys which is expected and in good agreement with the literature data [32,38]. At the same time kidney accumulation of [^68^Ga]**Ga-FA-II** is 2–3.5 times lower (*p* ≤ 0,01) than that of [^68^Ga]**Ga-FA-I**–Figure 4.

It is noteworthy that while [^68^Ga]**Ga-FA-I** biodistribution is characterized by a systematic decrease in kidney uptake over time, for [^68^Ga]**Ga-FA-II** this value practically does not change over time (30–120 min after administration) and equals 4.3 ± 1.1% ID/g (*p* > 0.05).

The content of both compounds in the blood was quite low and the difference between [^68^Ga]**Ga-FA-II** and [^68^Ga]**Ga-FA-I** was statistically insignificant (*p* > 0.05).

On average, the accumulation of [^68^Ga]**Ga-FA-II** in the liver and muscles after 30–120 min post injection was 2–3 times lower (*p* < 0.05) compared to that of [^68^Ga]**Ga-FA-I**. Thus, experimental data confirm the effectiveness of using HEHE tag in the structure of folic acid conjugates for reducing the accumulation in non-target organs and tissues. Blocking experiments (Table 2) indicate that for both labelled compounds kidney accumulation is FR-associated and decreases significantly (*p* < 0.001) with preliminary injection of folic acid. 

#### 2.4.2. Biodistribution in KB-tumor Murine Xenograft Models 

**Figure 5:**
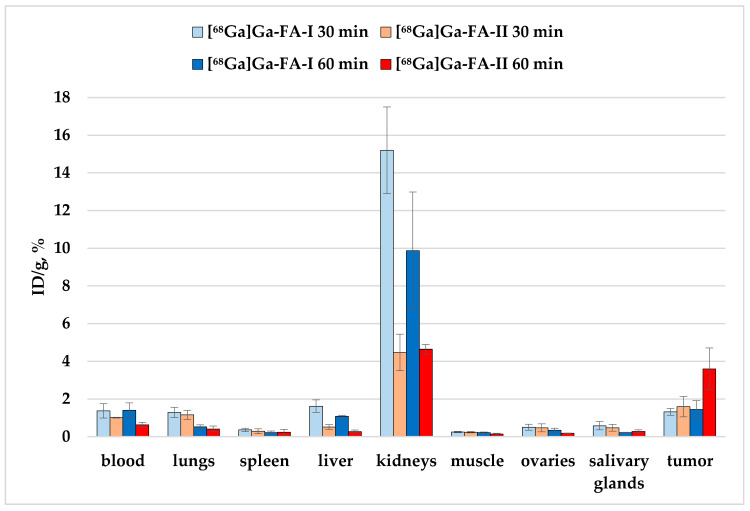
Biodistribution of [^68^Ga]**Ga-FA-I** and [^68^Ga]**Ga-FA-II** in KB-tumor bearing BALB/c nude mice 30 and 60 min after i.v. injection (selected organs/tissues from Table 3).

The results of the biodistribution study of [^68^Ga]**Ga-FA-I** and [^68^Ga]**Ga-FA-II** in BALB/c nude mice with KB xenografts are presented in Table 3 and Figure 5.

The experimental data show that in the case of mice with xenografts, the accumulation of [^68^Ga]**Ga-FA-II** for individual organs is either lower (for example, in kidneys and liver, *p* < 0.01) compared to [^68^Ga]**Ga-FA-I**, or does not change significantly (e.g., for lungs and stomach, *p* > 0.05).

The accumulation of [^68^Ga]**Ga-FA-I** and [^68^Ga]**Ga-FA-II** in the tumor site 30 min after injection is within the limits of experimental error: 1.32 ± 0.18% and 1.59 ± 0.54%, respectively (*p* > 0.05). The accumulation of [^68^Ga]**Ga-FA-I** 60 min after administration remained virtually the same as after 30 min (*p* > 0.05). But for [^68^Ga]**Ga-FA-II** an unexpected increase of the tumor uptake was observed: 60 min post injection it was 3.6 ± 1.1%, which is 2.5 times higher than that of [^68^Ga]**Ga-FA-I** by the same time point (*p* < 0.01).

**Figure 6:**
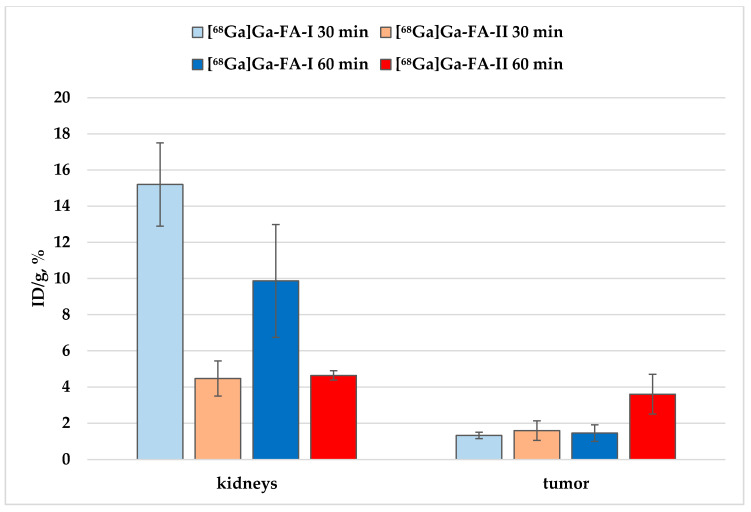
Accumulation of [^68^Ga]**Ga-FA-I** and [^68^Ga]**Ga-FA-II** in kidneys and KB-tumor xenografts.

As already mentioned, the experimental data (Figure 5) convincingly demonstrate that, with the exception of tumor foci, the accumulation of [^68^Ga]**Ga-FA-II** in the important organs and tissues is lower than that of [^68^Ga]**Ga-FA-I**. Thus, the tumor/healthy tissue ratio for [^68^Ga]**Ga-FA-II** is higher than that for [^68^Ga]**Ga-FA-I** (Table 4, *p* > 0,05 after 30 min, *p* < 0,01 after 60 min). The accumulation of both compounds in the kidneys was quite high and significantly exceeded the accumulation in the tumor foci (Figure 6).

However, it is seen that the accumulation of [^68^Ga]**Ga-FA-II** in the kidneys is significantly lower than that of [^68^Ga]**Ga-FA-I**:~3 times lower 30 min after administration (*p* < 0.001) and ~2 times lower 60 min after administration (*p* < 0.01). At the same time, the level of accumulation in the tumor foci was comparable (30 min, *p* > 0.05) for both labeled compounds, and even superior for [^68^Ga]**Ga-FA-II** in relation to [^68^Ga]**Ga-FA-I** after 60 min (*p* > 0.01). These results indicate an increase in potential imaging contrast when using [^68^Ga]**Ga-FA-II** due to the HEHE tag included in its structure.

During the biodistribution study of [^68^Ga]**Ga-FA-I** and [^68^Ga]**Ga-FA-II** in immunodeficient mice with transplanted KB tumors, one of the mice autopsy (at the time point of 30 min) revealed a spontaneous neoplasm in the groin area. This neoplasm was included in the layout of organs for measurement by direct radiometry. The measurement results are presented in Supplementary Materials (Table S3, Figure S16). 

## 3. Discussion

The high potential of folates for the molecular imaging of pathological processes has attracted the attention of many scientific groups in the world. To date, a lot of research has been reported on various folate-based conjugates with a variety of radionuclides. With all the attractive features of radiofolates for the purposes of radionuclide diagnostics and therapy, high level of accumulation in the kidneys is perhaps the main drawback that prevents an active development of this concept and its translation into clinical practice. Significant successes, however, have been achieved to date in solving this issue [25,39].

Relying on the impressive literature data [26,27,28,29,30] we attempted to neutralize high folate accumulation in kidney tissue by modifying the chemical structure of the conjugates introducing a histidine/glutamic acid tag. For the initial assessment of the suitability of this concept, we decided to start using doubled tag–(HE)_2_.

The results of in vitro studies with FR(+) and FR(−) cell lines along with blocking experiments showed that the accumulation of [^68^Ga]**Ga-FA-I** and [^68^Ga]**Ga-FA-II** in FR(+) cell cultures (KB, HeLa and HCT-116) is receptor-specific. Although in [26,27,28,29,30] it was shown that the level of receptor-specific binding often decreases when (HE)_n_-tag is used, in our studies the accumulation level of the modified and unmodified conjugates in the KB and HeLa cell cultures was almost the same. Only the profile of cell uptake over time is different for the two labelled compounds. The fact that [^68^Ga]**Ga-FA-II** has uptake in HCT-116 cells twice as high as that for [^68^Ga]**Ga-FA-I** is extremely unexpected and requires further study.

However, more important is the fact that [^68^Ga]**Ga-FA-I** uptake in the KB cells is significantly lower than that for the closest structural analogue published in the literature–^68^Ga-P3246 [32]. In Table 5 in vitro published data for folate-based ^68^Ga-conjugates [17,20,32,38,39,40] along with the molecules we synthesized are presented. Structures of these compounds are presented in Supplementary Materials for comparison (Figure S17).

Table 5 demonstrates that cell uptake for folate-based conjugates obtained by different groups varies widely. Thus, the accumulation of ^68^Ga-P3246 (**VI** in Figure S17–the most comparable analogue of [^68^Ga]**Ga-FA-I**) is almost 18 times higher than that obtained by us for [^68^Ga]**Ga-FA-I** (**VII** in Figure S17). Probably, the decisive factor here is that in [32] folate-free RMPI-1640 growth medium was used, while in this study EMEM (containing 1 mg/L of folic acid) was used. At the same time, two other similar in structure compounds–**III** and **V** (Figure S17) showed uptake level similar to [^68^Ga]**Ga-FA-I**, although the same folate-free RMPI-1640 medium was used when working with them. On the other hand, **III** and **V** contain NOTA chelator in their structure, which is similar but not equal to NODAGA used as chelator in **VI** and **VII**. And all four compounds (**III**, **V**, **VI** and **VII**) differ in the linker moieties as well. All of it affirms the significance of ‘linker-chelator’ fragment influence on receptor specificity of radiotracers. This effect was demonstrated earlier using ^68^Ga-labeled PSMA ligands [41,42]. Another possible explanation of the differences is related to the KB cell culture itself. The KB cell culture used by us was obtained from the collection of cell cultures of Ivanovsky Institute of Virology, where it was received in 1977 from the Engelhardt Institute of Molecular Biology of Russian Academy of Sciences, to which, in turn, it was provided by the author who first described it [43]. Unfortunately, the passage number of KB cells since that time is unknown. It is quite possible that this line degenerates, leading to a significant decrease in the number of folate receptors in it. Thus, in further studies, comparative experiments will be needed using this culture of KB cells and KB cultures from other cell collections (for example, ATCC). 

However, it seems the most probable that it is the diversity of experimental conditions and methodologies that lead to the differences presented in Table 6. Thus, the only data providing reliable comparison can be those obtained within one experiment. That is why to evaluate the effect of using (HE)_2_-tag both [^68^Ga]**Ga-FA-I** and [^68^Ga]**Ga-FA-II** where used in this study. Still in order to be able to compare our results with the other published data further research will apparently require bringing the methodology more in line with known trends, e.g., using folate-free media. At this stage of the study comparing the results for [^68^Ga]**Ga-FA-I** and [^68^Ga]**Ga-FA-II** we can conclude that the introduction of (HE)_2_-tag does not affect FR binding ability of the conjugates.

The results of the in vivo evaluation of [^68^Ga]**Ga-FA-I** and [^68^Ga]**Ga-FA-II** predictably showed that the introduction of (HE)_2_-tag results in 2–3.5 fold decrease of the kidney uptake. The accumulation of [^68^Ga]**Ga-FA-II** in the liver and muscles in the range from 30 to 120 min after administration was 2–3 times lower compared to [^68^Ga]**Ga-FA-I**. And the accumulation of both compounds in the blood did not differ significantly and was fairly low.

The accumulation of [^68^Ga]**Ga-FA-I** and [^68^Ga]**Ga-FA-II** in the tumor site (KB) 30 min after injection is similar: 1.32 ± 0.18% and 1.59 ± 0.54%, respectively. The tumor uptake of [^68^Ga]**Ga-FA-I** 60 min post injection did not change significantly. 2.5-fold increase of [^68^Ga]**Ga-FA-II** tumor uptake 60 min post injection was unexpected, but not unprecedented. Thus, in [30] it was shown by Liolios et al. that bispecific PSMA/GRPr conjugate containing (HE)_2_-tag showed significantly higher tumor/healthy tissue ratio in in vivo LNCaP tumor models than its (HE)_3_-, (HE)_1_- and (HE)_0_-tag analogues. In particular, the tumor/muscle ratio 60 min after administration was 2.48 ± 0.70, 6.03 ± 4.54 and 2.27 ± 1.45 for conjugates with (HE)_0_-, (HE)_1_- and (HE)_3_-tag respectively, and for (HE)_2_-tag conjugate this value was 22.65 ± 9.69, alongside with that during in vitro tests the accumulation of (HE)_2_-containing conjugate in LNCaP cells was almost three times lower than that for unmodified compound. In Table 6 the comparison of in vivo experimental and literature data is presented.

Table 6 demonstrates that kidney and tumor uptake levels obtained in the course of this study are the lowest. However, in most experiments analyzed animals were kept on folate-deficient diet. This fact probably explains the difference. This assumption is partly affirmed by the data presented in [39]. Unfortunately, at the time of the study, we were not able to reproduce these conditions, but this will be taken into consideration in further works. Thus, again, we can reliably compare only our data for the modified and unmodified compounds. And these data indicate that the introduction of (HE)_2_-tag into the structure does not affect the accumulation of the radiotracer in tumor negatively, but at the same time it significantly reduces the background accumulation. 

## 4. Materials and Methods

### 4.1. Chemicals and Reagents

Only deionized water 18.2 MΩ·cm (Milli-Q Millipore or TKA Smart2Pure) was used. All chemicals and solvents were of high-purity or pharmaceutical grade. The chemicals (including protected amino acids and 2-chlorotritylhloride resin (2-CTC resin)) were purchased from Sigma-Aldrich/Merck (St. Louis, MO, USA), Panreac Quimica (Barcelona, Spain) or abcr GmbH (Karlsruhe, Germany), unless otherwise indicated. NODAGA(tBu)_3_ precursor was purchased from CheMatech (Dijon, France).

### 4.2. ^68^Ge/^68^Ga Generator

^68^Ge/^68^Ga generator (Cyclotron Co., Ltd., Obninsk, Russia) with the initial activity of 1850 MBq was used. Post-processing of generator eluate was performed with HCl-ethanol method [44]. The final purified and concentrated solution of ^68^Ga was obtained in 0.1 M HCl_aq._ medium.

### 4.3. Synthesis of NODAGA-1,4-Buthanediamine--Folic Acid (***4*** in Scheme 1, ***FA-I***)

Synthesis of **FA-I** was carried out in four steps according to Scheme 1 based on the procedures described in [33] Short description of every step is presented below.

#### 4.3.1. Synthesis of N--BOC-1,4-Buthanediamine--Folic Acid (**1**)

Folic acid solution in DMSO (25 mL, 1.34 mmoL, 1 eq. dehydrated powder) was added to 308 mg (2 equiv.) of NHS and 552 mg (2 equiv.) of DCC. The reaction mixture was stirred for 16 h at RT, and then the urea precipitate was filtered off. TEA (0.376 mL, 2 equiv.) followed by N-Boc-1,4-butanediamine (575 mg, 2 equiv.) in DMSO (5 mL) were added to the filtrate. The mixture was stirred overnight and then added to a mixture of acetone and diethyl ether (1:4). The thin yellow precipitate was carefully centrifuged and washed with acetone and diethyl ether, and then dried under vacuum (530 mg, 65% yield). The identity of **1** was confirmed by NMR and HRMS (ESI) spectroscopy. ^1^H-NMR (400 MHz, DMSO-*d_6_*). δ 11.69 (br s, 1H, COOH), 8.62 (s, 1H, pterin), 8.04−7.93 (m, 1H), 7.85−7.75 (m, 1H), 7.70−7.55 (m, 2H, aromatic), 7.21−6.70 (m, 3H), 6.63 (d, 2H, *J* = 8.38, aromatic), 4.46 (bs, 2H, benzylic), 4.35–4.15 (m, 1H, αH), 3.05−2.80 (m, 5H, butanediamine), 2.35–1.77 (m, 4H, glutamic moiety),1.45–1.25 (m, 9H, Boc + CH_2_), 1.09 (t, 2H, *J* = 7.21, CH_2_). ^13^C-NMR (101 MHz, DMSO-*d_6_*) δ 174.41, 154.22, 150.78, 148.52, 148.42, 145.50, 128.87, 121.89, 121.60, 115.54, 111.26, 77.35, 69.65, 45.48, 32.14, 28.29, 26.98, 26.50, 8.91. NMR spectra are presented in the Supplementary Materials, Figures S1 and S2. HRMS (ESI) *m/z* 612.2901 (calcd 612.2889 for C_28_H_37_N_9_O_7_ [M + H]^+^); *m/z* 634.2725 (calcd 634.2725 for C_28_H_37_N_9_O_7_ [M + Na]^+^); *m/z* 650.2421 (calcd 650.2448 for C_28_H_37_N_9_O_7_ [M + K] ^+^ ). The HRMS (ESI) spectrum is presented in Supplementary Materials, Figure S5.

#### 4.3.2. Synthesis of 1,4-Butanediamine-Folic Acid (**2**)

Compound **1** (300 mg) was dissolved in TFA (3 mL) and stirred for two hours. The solvent was removed, and the residue was dissolved in the minimal amount of DMF. The addition of TEA resulted in the precipitation of a yellow residue which was centrifuged and washed with acetone and diethyl ether (228 mg, 91% yield). The identity of **2** was confirmed by NMR and HRMS (ESI) spectroscopy. ^1^H-NMR (400 MHz, DMSO-*d_6_*/D_2_O) δ 8.61 (s, 1H, pterin), 7.56 (m, 2H, *J* = 8.31, aromatic), 6.66−6.63 (m, 2H, J = 8.31, aromatic), 4.45 (s, 2H, benzylic), 4.18−4.05 (m, 1H, αH glutamic), 3.07−2.93 (m, 3H, butanediamine), 2.83−2.63 (m, 2H, butanediamine), 2.25−1.75 (m, 6H, glutamic moiety+ butanediamine), 1.59−1.26 (m, 3H, glutamic moiety+ butanediamine) 1.26−1.15 (m, 1H, glutamic moiety+ butanediamine), 1.15–1.08 (m, 3H, glutamic moiety + butanediamine). The NMR spectrum is presented in Supplementary Materials, Figure S3. HRMS (ESI) *m/z* 512.2369 (calcd 512.2364 for C_23_H_29_N_9_O_5_ [M + H]^+^); *m/z* 534.2190 (calcd. 534.2184 for C_23_H_29_N_9_O_5_ [M + Na]^+^). The HRMS (ESI) spectrum is presented in Supplementary Materials, Figure S6.

#### 4.3.3. Synthesis of NODAGA(tBu)_3–_1,4-Butanediamine-Folic Acid (**3**)

To a solution of NODAGA(tBu)_3_ (15 mg, 0.028 mmoL) in DMF EDC·HCl (3 mL, 6.6 mg, 0.034 mmoL) and NHS (3.8 mg, 0.033 mmoL) were added and stirred for 18 h at RT. Then 10 mL of dichloromethane was added to the solution followed by washing with brine (2 × 10 mL), 5% NaHCO_3_ (2 × 10 mL) and brine (1 × 10 mL). Washed organic fraction was dried under Na_2_SO_4_ and evaporated in vacuo. Obtained crude product was dissolved in 2 mL of DMF and then added to a solution of **2** (13 mg, 0.0248 mmoL) in 1 mL of DMF followed by addition of TEA (5 µL, 0.0345 mmoL). The solution was stirred overnight, then solvent was evaporated in vacuo. Crude product was purified by reverse phase chromatography (gradient CH_3_CN/H_2_O from 10% to 100% CH_3_CN, 25 min, flow rate–20 mL/min, 20g C18HP cartridge, 15 µm) giving 20 mg (77%) of desired product **3**. The identity of **1** was confirmed by HRMS (ESI) spectroscopy. HRMS (ESI) *m/z* 1037.5757 (calcd 1037.5778 for C_50_H_76_N_12_O_12_ [M + H]^+^). The HRMS (ESI) spectrum is presented in Supplementary Materials, Figure S7. Total purity of **3** was 100% as confirmed by LC-MS. Retention time is 11.59 min (method details are presented in the Supplementary Materials).

#### 4.3.4. Synthesis of NODAGA-1,4-Butanediamine-Folic Acid (**4, FA-I**)

A mixture of TFA/TIPS/H_2_O (2.5 mL; 95/2.5/2.5) was added to 10 mg (9.64 µmol) of **3** and stirred for 2 h at RT. The mixture was concentrated in vacuo and crude product was precipitated by addition of 5 mL of diethyl ether, centrifuged, and washed with acetone (2 × 2 mL), then diethyl ether diethyl ether (2 × 2 mL) before drying under vacuum, giving 6 mg (6.90 µmol) of compound **4** (**II**, 72%) which was identified by LC-MS and HRMS. LC-MS results: retention time: 9.18 min, total purity 91% (Supplementary Materials). HRMS (ESI) *m/z* 869.3906 (calcd. 869.3900 for C_38_H_52_N_12_O_12_ [M + H]^+^); *m/z* 891.3734 (calcd. 891.3720 for C_38_H_52_N_12_O_12_ [M + Na]^+^). The HRMS (ESI) spectrum is presented in Supplementary Materials, Figure S13. 

### 4.4. Synthesis of NODAGA-[Lys-(HE)_2_]--Folic Acid (***9*** in Scheme 2, ***FA-II***)

Synthesis of **FA-II** was carried out in four steps according to Scheme 2, based on the procedures described in [29,34,45]. Short descriptions of every step are presented below.

#### 4.4.1. Synthesis of Lys-(HE)_2_ Sequence Bound onto 2-CTC Resin (**5**)

The trityl- and *tert*-butyl and Boc-protected Lys-(HE)*_2_* sequence was synthesized according to standard Fmoc protocols using 2-CTC resin (319 mg) and the respective trityl- and *tert*-butyl–protected amino acids.

#### 4.4.2. Synthesis of [Lys-(HE)_2_]--Folic Acid Bound onto the Resin (**6**)

Folic acid (890 mg, 2.02 mmoL) and diisopropylethylamine (423 mL, 2.42 mmoL) were dissolved in DMSO (7 mL) preheated to 50 °C. Then resin was added followed by PyBOP (1050 mg, 2.02 mmoL) and the mixture was stirred overnight at RT. The mixture was filtered and the resin washed with DMSO (3 × 10 mL), *N*,*N*-dimethylformamide (3 × 10 mL), dichloromethane (5 × 10 mL) and methanol (5 × 10 mL) resulting in an orange resin (1.063 g).

#### 4.4.3. Cleavage of [Lys-(HE)_2_]-Folic Acid from the Resin (**7**)

A mixture of TFA/TIPS/H_2_O (7 mL; 95/2.5/2.5) was added to the resin (300 mg) and stirred for 2 h at RT. The mixture was filtered, and the resin washed with dichloromethane (10 mL). Organic fraction was concentrated in vacuo. The crude product was precipitated by addition of 10 mL of diethyl ether, centrifuged, and washed with acetone (2 × 4 mL), then diethyl ether diethyl ether (2 × 4 mL) before drying under vaccum. 116 mg of **7** was obtained (52%) as orange powder which was identified with ^1^H-NMR, HRMS (ESI) and LC-MS [34,46]. ^1^H-NMR (400 MHz, DMSO-*d*_6_) δ 8.63 (d, 2H, J = 12.53), 8.63 (s, 1H, pterin), 8.45–7.95 (m, 7H, Ar + NH), 7.92–7.72 (m, 3H, Ar + NH), 8.71–7.55 (m, 2H, Ar + NH), 7.33–7.19 (m, 3H, Ar), 7.19–6.89 (m, 2H, Ar), 6.63 (d, 2H, J = 8.13, aromatic), 4.67–4.50 (m, 3H, αCH), 4.47 (s, 2H, benzylic), 4.39–4.25 (m, 1H, CH), 4.25–4.05 (m, 4H, CH), 3.23–2.84 (m, 2H, CH_2_), 2.79–2.62 (m, 3H, CH_2_), 2.41–2.12 (m, 8H, CH_2_), 2.02–1.62 (m, 8H, CH_2_), 1.62–1.36 (m, 5H, CH_2_), 1.36–1.12 (m, 3H, CH_2_). The NMR spectrum is presented in the Supplementary Materials, Figure S4. HRMS (ESI) *m/z* 1100.4305 (calcd 1100.4293 for C_47_H_59_N_17_O_15_ [M−H]^−^); *m/z* 1122.4130 (calcd 1122.4112 for C_47_H_59_N_17_O_15_ [M-2H + Na]^−^). The HRMS (ESI) spectrum is presented in Supplementary Materials, Figure S9. LC-MS results: retention time: 8.53 min, total purity 92% (Supplementary Materials).

#### 4.4.4. Synthesis of NODAGA(tBu)_3_-[Lys-(HE)_2_]-Folic Acid (**8**)

To a solution of NODAGA(tBu)_3_ (15 mg, 0.028 mmoL) in DMF (3 mL) EDC*HCl (6.6 mg, 0.034 mmoL) and NHS (3.8 mg, 0.033 mmoL) were added and stirred for 18 h at RT. Then 10 mL of dichloromethane was added to the solution followed by washing with brine (2 × 10 mL), 5% NaHCO_3_ (2 × 10 mL) and brine (1 × 10 mL). Washed organic fraction was dried under Na_2_SO_4_ and evaporated in vacuo. Obtained crude product was dissolved in 2 mL of DMF, subsequently solution of **7** (42 mg, 0.0248 mmoL) in 1 mL of DMF was added followed by addition of DIPEA (60 µL, 0.345 mmoL). The solution was stirred overnight, then solvent was evaporated in vacuo. Crude product was purified by reverse phase chromatography (gradient CH_3_CN/H_2_O from 10% to 100% CH_3_CN, 25 min, flow rate–20 mL/min, 20g C18HP cartridge, 15 µm) giving 10 mg (25%) of desired product **8**. The identity and purity were confirmed with HRMS (ESI) and LC-MS. HRMS (ESI) *m/z* 1625.7672 (calcd 1625.7707 for C_74_H_106_N_20_O_22_ [M−H]^−^). The HRMS (ESI) spectrum is presented in the Supplementary Materials, Figure S10. LC-MS results: retention time: 10.51 min, total purity 99% (Supplementary Materials).

#### 4.4.5. Synthesis of NODAGA-[Lys-(HE)_2_]-Folic Acid (**9, FA-II**)

A mixture of TFA/TIPS/H_2_O (3 mL; 95/2.5/2.5) was added to 4 mg (2.46 µmol) of **8** and stirred for 2 h at RT. The mixture was concentrated in vacuo and crude product was precipitated by addition of 5 mL of diethyl ether, centrifuged, and washed with acetone (2 × 2 mL), then diethyl ether (2 × 2 mL) before drying under vacuum, giving 3.1 mg (2.12 µmol) of compound **9** (86%) which was identified by LC-MS and HRMS. HRMS (ESI) *m/z* 728.2887 (calcd 728.2871 for C_62_H_82_N_20_O_22_ [M-2H]^2−^); *m/z* 1457.5856 (calcd. 1457.5829 for C_62_H_82_N_20_O_22_ [M−H]^−^). The HRMS (ESI) spectrum is presented in the Supplementary Materials, Figure S11. LC-MS results: retention time: 8.827 min, total purity 94% (Supplementary Materials).

### 4.5. Preparation and Quality Control of ^68^Ga-Labeled Compounds

[^68^Ga]**Ga-FA-I** and [^68^Ga]**Ga-FA-II** were obtained using standard procedures described in [47]. In short, to 2.0 mL Eppendorf test tube containing various amounts of precursor (10–20 μg), an aqueous solution of sodium acetate (0.3 M, 400–500 μL) and conditioned (purified and concentrated) eluate of ^68^Ge/^68^Ga generator (400–500 μL) were added. For pH adjustment 2 M HCl (~ 90 μL) was used. The reaction mixtures were incubated at 25 °C for 10–15 min. The activity of each preparation was from 50 to 250 MBq, pH 4.5–5.

A number of radio-TLC and radio-HPLC methods were used to analyze the radiochemical purity of preparations obtained [45]. The main TLC methods were: ITLC-SG paper (Thermo Fisher Scientific, Waltham, MA, USA) with 4% TFA in water (*v/v*), 0.05 M citric acid water solution, 1 M CH_3_COONH_4_ in methanol–water mixture (1:1) as solvents [45]. For HPLC two methods described in [48] were used. In short: Knauer Smartline HPLC system (Berlin, Germany) equipped with an fLumo radiometric detector (Berthold, Bad Wildbad, Germany) and reversed-phase C18 columns was used. The column thermostat temperature was 40 °C. Method 1: 250 × 4.6 mm column (Jupiter, Phenomenex Inc., Torrance, CA, USA); isocratic flow (1.0 mL min^−1^): 25% of 0.1% TFA in water, 75% of acetonitrile. Retention times using Method 1 for [^68^Ga]**Ga-FA-I** and [^68^Ga]**Ga-FA-II** were 9.4 ± 0.2 and 11.4 ± 0.2 min correspondingly. Method 2: 150 × 4.6 mm column (Advanced Chromatography Technologies Ltd., Aberdeen, UK) gradient flow (1.2 mL min^−1^): 0–3–6–8–9–15 min = 100–100–0–0–100–100% A (A – 0.05 M citric acid in water, B–acetonitrile). Retention times using Method 2 for [^68^Ga]**Ga-FA-I** and [^68^Ga]**Ga-FA-II** were 6.5 ± 0.2 and 5.5 ± 0.2 min correspondingly. 

Examples of TLC- and HPLC-chromatograms obtained are presented in Supplementary Materials (Table S1, Figure S14). When needed (e.g., if peaks other than unbound/colloidal ^68^Ga were observed, see Supplementary Materials, Table S2, Figure S15) after labeling reaction and QC the preparations were reformulated into saline media using C_18_ Sep Pak cartridges (Waters).

Radiochemical purity of all [^68^Ga]**Ga-FA-I** and [^68^Ga]**Ga-FA-II** final preparations used for biological studies was ≥ 98%.

### 4.6. n-Octanol/Water Distribution Coefficient (LogD_ow_ Value)

The distribution coefficients (log*D*_ow_ values) of the [^68^Ga]**Ga-FA-I** and [^68^Ga]**Ga-FA-II** were determined using standard procedure [49]. In brief, samples containing ∼ 3.5 MBq of [^68^Ga]**Ga-FA-I** and [^68^Ga]**Ga-FA-II** in a volume of 50 μL were added to each vial containing 1450 μL of water and 1500 μL of pre-saturated n-octanol. The vials were vortexed vigorously for 5 min and then stored (5 min) for phase separation. The concentration of radioactivity in a defined volume of each layer was measured using a WIZARD^2^ automatic γ-counter (PerkinElmer, Waltham, MA, USA). The distribution coefficient was expressed as the logarithm of the ratio of counts per minute (cpm) measured in the n-octanol phase to the cpm measured in the water phase. The values reported are the means of 3−5 independent measurements ( ± SD), each performed with 5 replicates.

### 4.7. Cell Cultures and In Vitro Studies

The human epithelial FR(+) cancer cell lines KB (cervical, collection of cell cultures of Ivanovsky Institute of Virology), HeLa (cervical, Biolot Ltd., Moscow, Russia), and HCT-116 (colorectal, ATCC CCL-247), and FR(−) A549 human lung cancer cell line (Biolot Ltd.) were used for in vitro receptor binding studies. The cells were cultured continuously as monolayers at 37 °C in a humidified atmosphere containing 5% CO_2_, in Eagle’s minimal essential medium (EMEM, Biolot Ltd.) supplemented with 10% fetal calf serum (FCS), L-glutamine, and antibiotic (streptomycin, 100 μg/mL). For in vitro and in vivo experiments, subconfluent cells were harvested by treatment with trypsin (0.05%) and EDTA (0.02%) in PBS.

For in vitro tests, the cells were seeded into six-well plates (1 × 10^6^ cells/well, 99% viability) and incubated at 37 °C/5% CO_2_ to form subconfluent monolayers overnight. The day of the experiment, the medium was removed, and the cells were washed twice with HBSS (no calcium, no magnesium, no phenol red). 900 μL of saline was added to each well, and the plates were incubated at 37 °C/5% CO_2_ for 1 h. After 1 h the radiotracer (0.5 nmol, 100 μL; 500 nM) in saline was added to the dedicated well. For blocking studies, cells were pre-incubated with an excess of folic acid (50 μM) 15 min prior to the addition of radiotracer. Experiments were performed in triplicate for each time point. After necessary time of incubation at 37 °C, the supernatant of each well was collected together with washing solution (ice-cold PBS, 2 × 1.0 mL). Then, cells were treated with 1 M sodium hydroxide solution (1.0 mL) for 10 min at RT. Finally, cell lysate was collected together with washing solution (ice-cold PBS, 2 × 1.0 mL). The radioactivity of all contents including initially added radiotracer was measured using a WIZARD^2^ automatic γ-counter (PerkinElmer). Receptor-specific uptake was calculated by cell-bound activity and expressed as a percentage of applied activity per million cells.

### 4.8. Biodistribution Studies

All experiments involving animals were performed following the ethical standards, Russian animal protection laws and guidelines for scientific animal trials [GOST 33216–2014 Guidelines for accommodation and care of animals. Species-specific provisions for laboratory rodents and rabbits] with permission of Ethical board of State Research Center−Burnasyan Federal Medical Biophysical Center of Federal Medical Biological Agency (decision №9б 11.08.2019).

Normal biodistribution studies were carried out in healthy Wistar rats. Animals were grouped (N = 5/group) and injected with 10−12 MBq/100 μL of each ^68^Ga-NODAGA-folate conjugate into the tail vein. Nonspecific uptake in FR-positive organs (e.g., kidneys) was determined by 5 min of folic acid pre-injection (36 μM, 100 μL) in PBS pH 7.4.

For the experimental tumor models athymic female BALB/c nude mice (4−5 weeks old, 18−20 g) were subcutaneously inoculated in the right flank with suspended 2 × 10^6^ KB cells (150 μL, Opti-MEM (Gibco, Thermo Fisher Scientific, Waltham, MA, USA) and 50% Matrigel (Becton Dickinson, Franklin Lakes, NJ, USA). Tumors were allowed to grow for 12−16 days (tumor weight 500−900 mg). Animals were grouped (N = 3–5/group) and injected with 10−12 MBq/100 μL of each ^68^Ga-NODAGA-folate conjugate into the tail vein.

At preselected time points of (30, 60, 90, 120 min for rats and 30, 60 min for mice) animals were sacrificed. The organs of interest were collected, blotted dry, weighed, and counted using a WIZARD^2^ automatic γ-counter (PerkinElmer). The results are expressed as the percentage of injected activity dose per gram (% ID/g ± SD) for each organ/tissue.

### 4.9. Statistical Analysis

Data were analyzed for significance using unpaired two-tailed t-test with Prism software (Prism software, version 6.05, GraphPad, San Diego, CA, USA). A *p*-value of ≤ 0.05 was considered statistically significant. 

## 5. Conclusions

At this point we can conclude that, within the framework of the conditions and results of this study, it was shown that the introduction of the HEHE tag into the structure of ^68^Ga-conjugates based on folic acid can significantly reduce the accumulation of these radiotracers in intact tissues and important organs, keeping the accumulation in tumor at least on the same level, and possibly increasing it. The study is ongoing. More detailed studies of folate-based ^68^Ga-conjugates, including both the effect of number of HE moieties (n) and the position of the tag in conjugate structure, as well as study of multimerization effect [31,50,51], appear to be very interesting and relevant.

## Figures and Tables

**Table 1:** Biodistribution of [^68^Ga]**Ga-FA-I** and [^68^Ga]**Ga-FA-II** in normal Wistar rats (*mean ± SD, n = 5*).

	ID/g,%							
	30 min		60 min		90 min		120 min	
Tissue/ Organ	[^68^Ga]**Ga-FA-I**	[^68^Ga]**Ga-FA-II**	[^68^Ga]**Ga-FA-I**	[^68^Ga]**Ga-FA-II**	[^68^Ga]**Ga-FA-I**	[^68^Ga]**Ga-FA-II**	[^68^Ga]**Ga-FA-I**	[^68^Ga]**Ga-FA-II**
blood	0.41 ± 0.10	0.28 ± 0.03	0.26 ± 0.07	0.22 ± 0.06	0.13 ± 0.03	0.09 ± 0.01	0.08 ± 0.02	0.06 ± 0.02
lungs	0.46 ± 0.08	0.28 ± 0.06	0.32 ± 0.09	0.24 ± 0.07	0.21 ± 0.08	0.12 ± 0.02	0.24 ± 0.02	0.11 ± 0.02
heart	0.30 ± 0.11	0.27 ± 0.07	0.28 ± 0.09	0.16 ± 0.08	0.18 ± 0.12	0.10 ± 0.01	0.12 ± 0.02	0.08 ± 0.01
kidneys	14.05 ± 4.35	4.03 ± 0.94	9.97 ± 1.28	4.64 ± 1.80	8.07 ± 1.41	4.42 ± 1.27	13.15 ± 1.11	4.34 ± 0.18
liver	0.40 ± 0.09	0.18 ± 0.06	0.25 ± 0.05	0.12 ± 0.06	0.19 ± 0.10	0.07 ± 0.01	0.16 ± 0.03	0.05 ± 0.01
stomach	0.17 ± 0.02	0.20 ± 0.02	0.14 ± 0.08	0.12 ± 0.05	0.17 ± 0.04	0.11 ± 0.01	0.14 ± 0.05	0.08 ± 0.04
spleen	0.16 ± 0.10	0.13 ± 0.04	0.09 ± 0.01	0.07 ± 0.04	0.06 ± 0.02	0.06 ± 0.02	0.09 ± 0.04	0.04 ± 0.02
intestines	0.37 ± 0.13	0.08 ± 0.01	0.26 ± 0.08	0.08 ± 0.02	0.31 ± 0.07	0.07 ± 0.01	0.32 ± 0.16	0.12 ± 0.08
bladder	0.27 ± 0.15	0.89 ± 0.80	0.36 ± 0.31	0.40 ± 0.16	0.16 ± 0.01	0.27 ± 0.03	0.16 ± 0.06	0.14 ± 0.02
muscle	0.29 ± 0.06	0.10 ± 0.03	0.12 ± 0.05	0.06 ± 0.01	0.14 ± 0.05	0.04 ± 0.01	0.11 ± 0.03	0.04 ± 0.01
brain	0.06 ± 0.03	0.04 ± 0.01	0.04 ± 0.01	0.02 ± 0.01	0.03 ± 0.01	0.02 ± 0.01	0.03 ± 0.01	0.01 ± 0.01

**Table 2:** Accumulation of [^68^Ga]**Ga-FA-I** and [^68^Ga]**Ga-FA-II** in the kidneys * of normal Wistar rats (*mean ± SD, n = 3*) 60 min after i.v. injection with/without folic acid pre-injection.

	ID/g,%			
**Organ**	[^68^Ga]**Ga-FA-I**	[^68^Ga]**Ga-FA-I** *with Folic Acid Pre-Injection*	[^68^Ga]**Ga-FA-II**	[^68^Ga]**Ga-FA-II** *with Folic Acid Pre-Injection*
kidneys	10.2 ± 1.3	1.05 ± 0.20	4.27 ± 0.93	1.26 ± 0.24

* other organs and tissues data are omitted for clarity due to low accumulation similar to that presented in Table 1.

**Table 3:** Biodistribution of [^68^Ga]**Ga-FA-I** and [^68^Ga]**Ga-FA-II** in KB-tumor bearing BALB/c nude mice (*mean ± SD, n = 5*).

	ID/g,%			
	30 min		60 min	
	[^68^Ga]Ga-FA-I	[^68^Ga]Ga-FA-II	[^68^Ga]Ga-FA-I	[^68^Ga]Ga-FA-II
blood	1.37 ± 0.38	1.01 ± 0.02	1.40 ± 0.39	0.63 ± 0.13
lungs	1.29 ± 0.27	1.16 ± 0.24	0.52 ± 0.11	0.41 ± 0.15
heart	0.97 ± 0.07	0.62 ± 0.13	0.79 ± 0.19	0.31 ± 0.13
stomach	0.61 ± 0.22	0.47 ± 0.17	0.59 ± 0.26	0.58 ± 0.30
spleen	0.36 ± 0.09	0.29 ± 0.13	0.22 ± 0.08	0.24 ± 0.14
liver	1.62 ± 0.33	0.51 ± 0.13	1.08 ± 0.06	0.27 ± 0.08
kidneys	15.2 ± 2.3	4.47 ± 0.97	9.87 ± 3.12	4.64 ± 0.26
bladder	7.7 ± 2.9	1.46 ± 0.53	2.6 ± 1.8	0.96 ± 0.46
intestines	1.21 ± 0.42	0.46 ± 0.15	1.03 ± 0.13	0.45 ± 0.13
brain	0.14 ± 0.08	0.09 ± 0.03	0.11 ± 0.01	0.10 ± 0.03
muscle	0.25 ± 0.03	0.24 ± 0.04	0.22 ± 0.03	0.14 ± 0.04
ovaries	0.50 ± 0.16	0.47 ± 0.21	0.34 ± 0.11	0.18 ± 0.01
salivary glands	0.58 ± 0.22	0.47 ± 0.18	0.22 ± 0.02	0.28 ± 0.08
tumor	1.32 ± 0.18	1.59 ± 0.54	1.45 ± 0.46	3.6 ± 1.1

**Table 4:** Differential uptake ratio (DUR) for [^68^Ga]**Ga-FA-I** and [^68^Ga]**Ga-FA-II** 30 and 60 min post injection in KB-tumor xenografts (*mean ± SD, n = 5*).

T/N	DUR			
	30 min		60 min	
	[^68^Ga]Ga-FA-I	[^68^Ga]Ga-FA-II	[^68^Ga]Ga-FA-I	[^68^Ga]Ga-FA-II
Tumor-to-kidneys	0.09 ± 0.03	0.39 ± 0.18	0.17 ± 0.11	0.79 ± 0.28
Tumor-to-muscle	5.4 ± 1.1	7.1 ± 2.8	6.9 ± 2.5	28.9 ± 13.7
Tumor-to-blood	1.0 ± 0.4	1.6 ± 0.5	1.2 ± 0.6	6.1 ± 2.5

**Table 5:** Results of in vitro binding assays with KB cells for folate-based conjugates.

Molecule *	Cells Per Well	Ligand Amount	Culture Medium	Incubation Temp, °C	Incubation Time, min	Cell-Associated Activity,%	Ref.
**I**	∼0.5–1.0 × 10^6^	∼35 pmol	Folate-free RPMI-1640	37	30	15–17	[17]
**II**	0.8–1.0 × 10^6^	2.5 pmol	Folate-free RPMI-1640	37	30–240	60–80	[38]
**III**	∼1.0 × 10^6^	0.15 μM^§^	Folate-free RPMI-1640	4	60	1.7	[39]
**IV**	∼1.0 × 10^5^	not specified	DMEM	37	30–120	0.25–0.91	[20]
**V**	∼1.0 × 10^6^	0.1 nmol	Folate-free RPMI-1640	37	30	2.84	[40]
**VI**	∼1.0 × 10^6^	2.5 pmol	Folate-free RPMI-1640	37	30–120	60–72	[32]
**VII**	∼1.0 × 10^6^	0.5 nmol	EMEM	37	30–90	2.9–3.9	This work
**VIII**	∼1.0 × 10^6^	0.5 nmol	EMEM	37	30–90	1.5–4.1	

* for chemical structures see Figure S17. ^§^ the authors present only ligand concentration per well.

**Table 6:** Biodistribution results for folate-based conjugates labeled with ^68^Ga.

Molecule *	Time After Injection, h	ID/g,%				Animals Were on Folate-Deficient Diet	Ref.
		Kidneys	Liver	Blood	KB Tumor		
**I**	1	19.20 ± 2.59	7.52 ± 0.55	12.76 ± 1.53	9.57 ± 0.97	+	[17]
	4	25.23 ± 2.40	4.20 ± 1.04	6.03 ± 1.11	11.92 ± 1.68		
**II**	1	82.21 ± 5.53	4.88 ± 0.81	0.32 ± 0.03	11.77 ± 2.76	+	[38]
	4	103.01 ± 24.58	2.08 ± 0.08	0.20 ± 0.04	14.29 ± 4.14		
**III**	1	15.07 ± 4.1	2.07 ± 0.6	1.17 ± 0.3	–	–	[39]
IV**	–	–	–	–	–	+	[20]
**V**	4	21.65 ± 1.11	0.38 ± 0.08	0.07 ± 0.01	6.61 ± 1.07	+	[40]
**VI**	1	91.52 ± 21.05	2.58 ± 0.26	0.24 ± 0.05	16.56 ± 3.67	+	[32]
	4	130.31 ± 14.65	1.07 ± 0.18	0.06 ± 0.01	16.29 ± 4.46		
**VII**	1	9.87 ± 3.12	1.08 ± 0.06	1.40 ± 0.39	1.45 ± 0.46	–	This work
**VIII**	1	4.64 ± 0.26	0.27 ± 0.08	0.63 ± 0.13	3.6 ± 1.1	–	

* for chemical structure see Figure S17. ** in vivo biodistribution was evaluated via μPET.

## Supplementary Materials

The following are available online. Figure S1: ^1^H NMR spectrum (400 MHz) of **1** in DMSO-*d*_6_, Figure S2: ^13^C NMR spectrum (101 MHz) of **1** in CDCl_3_, Figure S3: ^1^H NMR spectrum (400 MHz) of **2** in DMSO-*d*_6_/D_2_O, Figure S4: ^1^H NMR spectrum (400 MHz) of **7** in DMSO-*d*_6_, Figure S5: HRMS (ESI) spectrum of **1**, Figure S6: HRMS (ESI) spectrum of **2**, Figure S7: HRMS (ESI) spectrum of **3**, Figure S8: HRMS (ESI) spectrum of **4**, Figure S9: HRMS (ESI) spectrum of **7**, Figure S10: HRMS (ESI) spectrum of **8**, Figure S11: HRMS (ESI) spectrum of **9**, Figure S12: LC-MS chromatogram of **4 (FA-I)**, Figure S13: LC-MS chromatogram of **9 (FA-II)**, Table S1: Radio-TLC chromatograms of [^68^Ga]**Ga-FA-I** and [^68^Ga]**Ga-FA-II**, Table S2: Examples of radio-TLC chromatograms pre- and post-reformulation, Table S3: Biodistribution of [^68^Ga]**Ga-FA-I** in KB-tumor bearing BALB/c nude mouse with spontaneous neoplasm 30 min after injection, Figure S16: Micrographs of spontaneous neoplasm from the groin of the mice at various magnifications. The histological picture corresponds to epidermal cyst of the skin with signs of suppuration of the wall, Figure S17: Chemical structure of [^68^Ga]**Ga-FA-I** (**VII**) and [^68^Ga]**Ga-FA-II** (**VIII**) in comparison with other published folate-based conjugates tested with radiogallium.
