# Supplementary material for: Pharmacokinetic Properties of 68Ga-labelled Folic Acid Conjugates: Improvement Using HEHE Tag
Source: Molecules. 2020 Jun 11;25(11):2712. doi: 10.3390/molecules25112712 (PMC7321154; doi:10.3390/molecules25112712)
Supplement: Supplementary file 1 [file molecules-25-02712-s001.pdf]

molecules

MDPI

Supplementary materials for:

Pharmacokinetic Properties of ^{68}Ga-labelled Folic Acid Conjugates: Improvement Using HEHE Purification Tag

Anton Larenkov, Marat Rakhimov, Kristina Lunyoa, Olga Klementyeva, Alesya Maruk, Aleksei Machulkin

Content:

**NMR**	Experiment details………………………..…………………………………………	2
**Figure S1.**	^{1}H NMR spectrum (400 MHz) of **1** in DMSO-d6……………………………………	2
**Figure S2.**	^{13}C NMR spectrum (101 MHz) of **1** in $CDCl_3$………………………………………	2
**Figure S3.**	^{1}H NMR spectrum (400 MHz) of **1** in DMSO-d6/D_2O……………………………	3
**Figure S4.**	^{1}H NMR spectrum (400 MHz) of **7** in DMSO-d6……………………………………	3
**HRMS (ESI)**	Experiment details………………………..…………………………………………	4
**Figure S5.**	HRMS (ESI) spectrum of **1**…………………..……………………………………..	4
**Figure S6.**	HRMS (ESI) spectrum of **2**…………………..…………………………………….	4
**Figure S7.**	HRMS (ESI) spectrum of **3**…………………..……………………………………..	5
**Figure S8.**	HRMS (ESI) spectrum of **4**…………………..……………………………………..	5
**Figure S9.**	HRMS (ESI) spectrum of **7**…………………..……………………………………..	6
**Figure S10.**	HRMS (ESI) spectrum of **8**…………………..……………………………………..	6
**Figure S11.**	HRMS (ESI) spectrum of **9**…………………..……………………………………..	7
**HPLC-MS**	Experiment details………………………..…………………………………………	8
**Figure S12.**	LC-MS chromatogram of **4 (FA-I)**……………………………………………….	8
**Figure S13.**	LC-MS chromatogram of **9 (FA-II)**………………………………………………	9
**Radio-TLC**		
**Table S1.**	Radio-TLC chromatograms of [^{68}Ga]Ga-FA-I and [^{68}Ga]Ga-FA-II………………...	10
**Table S2.**	Examples of radio-TLC chromatograms pre- and post-reformulation…………	11
**Radio-HPLC**		
**Figure S14.**	HPLC chromatograms obtained with Method 1 for [^{68}Ga]Ga-FA-I and [^{68}Ga]Ga-FA-II, and with Method 2 for [^{68}Ga]Ga-FA-I and [^{68}Ga]Ga-FA-II………	12
**Figure S15.**	HPLC chromatograms obtained with Method 1 for [^{68}Ga]Ga-FA-I and [^{68}Ga]Ga-FA-II pre- and post-reformulation……………………………………………	12
**The case of [^{68}Ga]Ga-FA-I accumulation in non-tumor neoplasm (cyst)**……………………………		13
**Table S3.**	Biodistribution of [^{68}Ga]**Ga-FA-I** in KB-tumor bearing BALB/c nude mouse with spontaneous neoplasm 30 min after injection………………………………………..	13
**Figure S16.**	Micrographs of spontaneous neoplasm from the groin of the mice at various magnifications. The histological picture corresponds to epidermal cyst of the skin with signs of suppuration of the wall………………………………………....	13
**Figure S17.**	Chemical structure of [^{68}Ga]**Ga-FA-I** (**VII**) and [^{68}Ga]**Ga-FA-II** (**VIII**) in comparison with other published folate-based conjugates tested with radiogallium…………………………………………………………………….	14

NMR

Experiment details

^1H and ^{13}C NMR spectra were registered on a Bruker Avance 400 spectrometer (400 MHz for ^1H and 90 MHz for ^{13}C) in $CDCl_3$ or DMSO-d6.

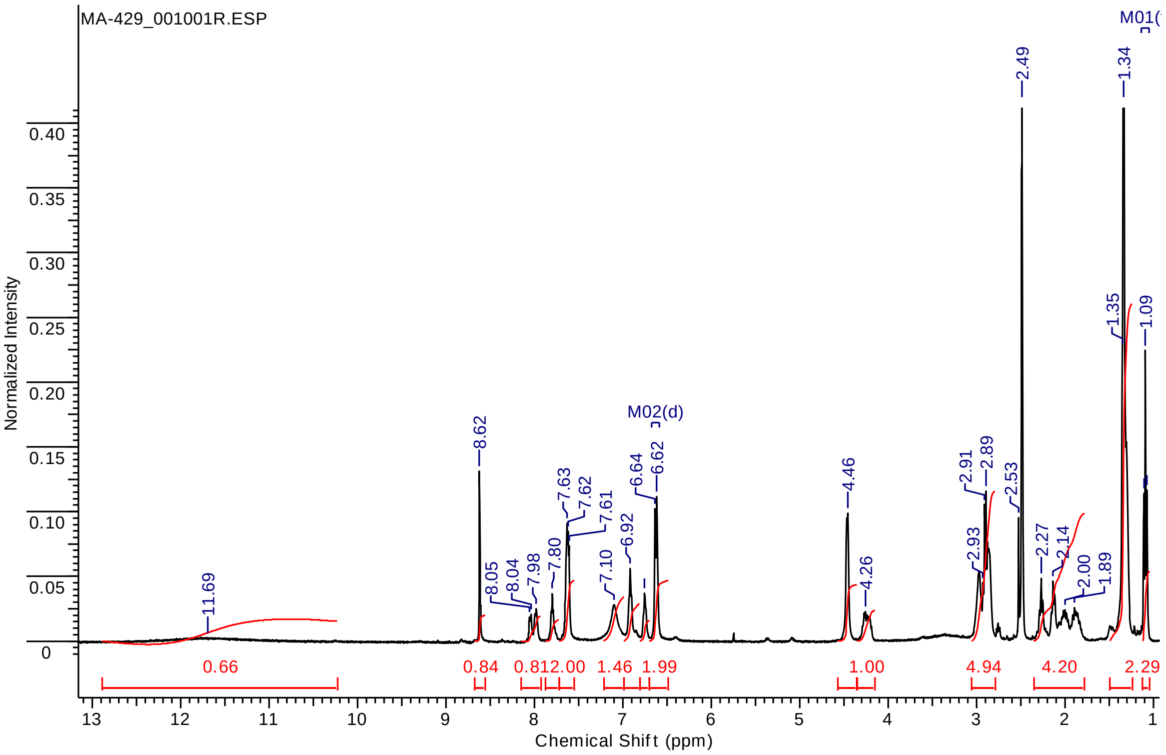

Figure S1. ^1H NMR spectrum (400 MHz) of **1** in DMSO-d6

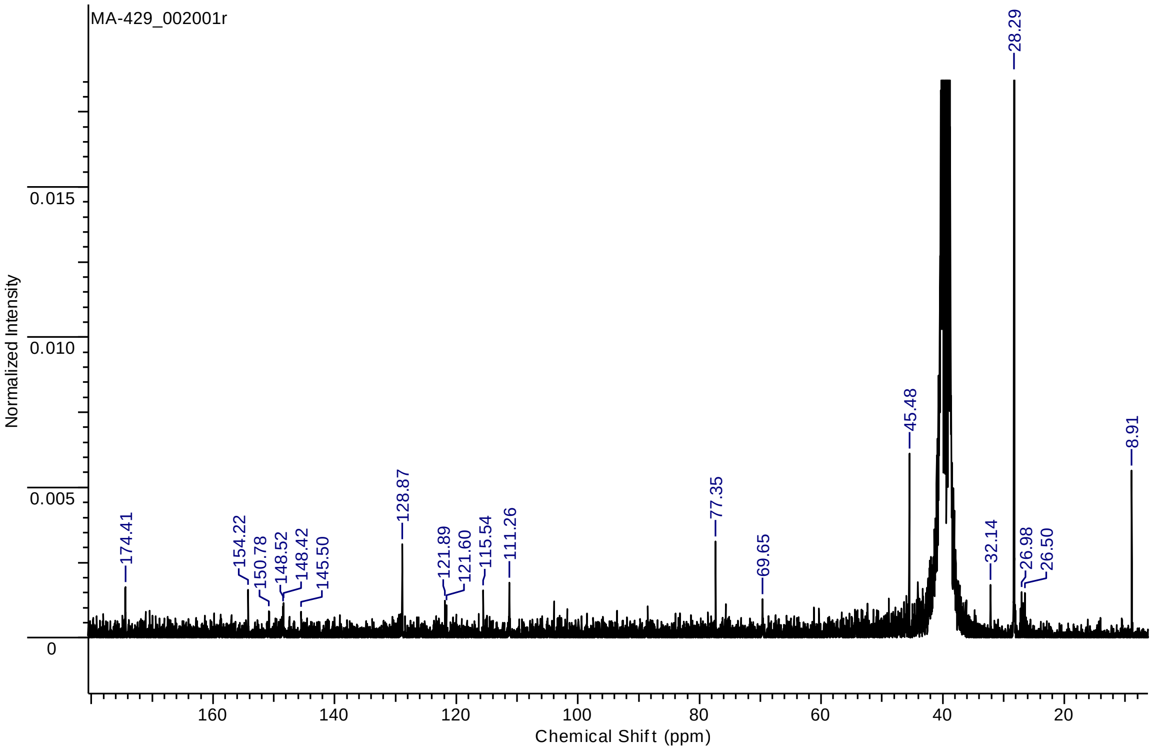

Figure S2. ^{13}C NMR spectrum (101 MHz) of **1** in $CDCl_3$

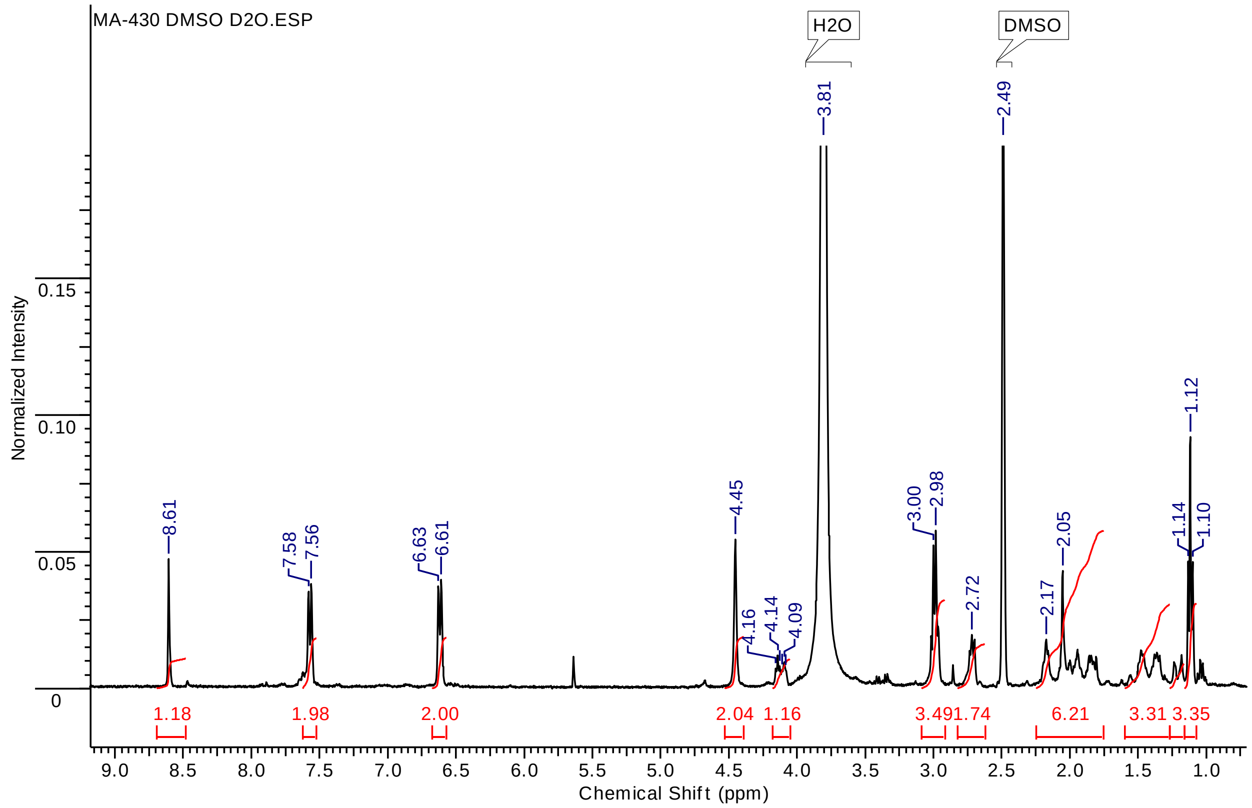

Figure S3. ^{1}H NMR spectrum (400 MHz) of **1** in DMSO-d6/D_2O

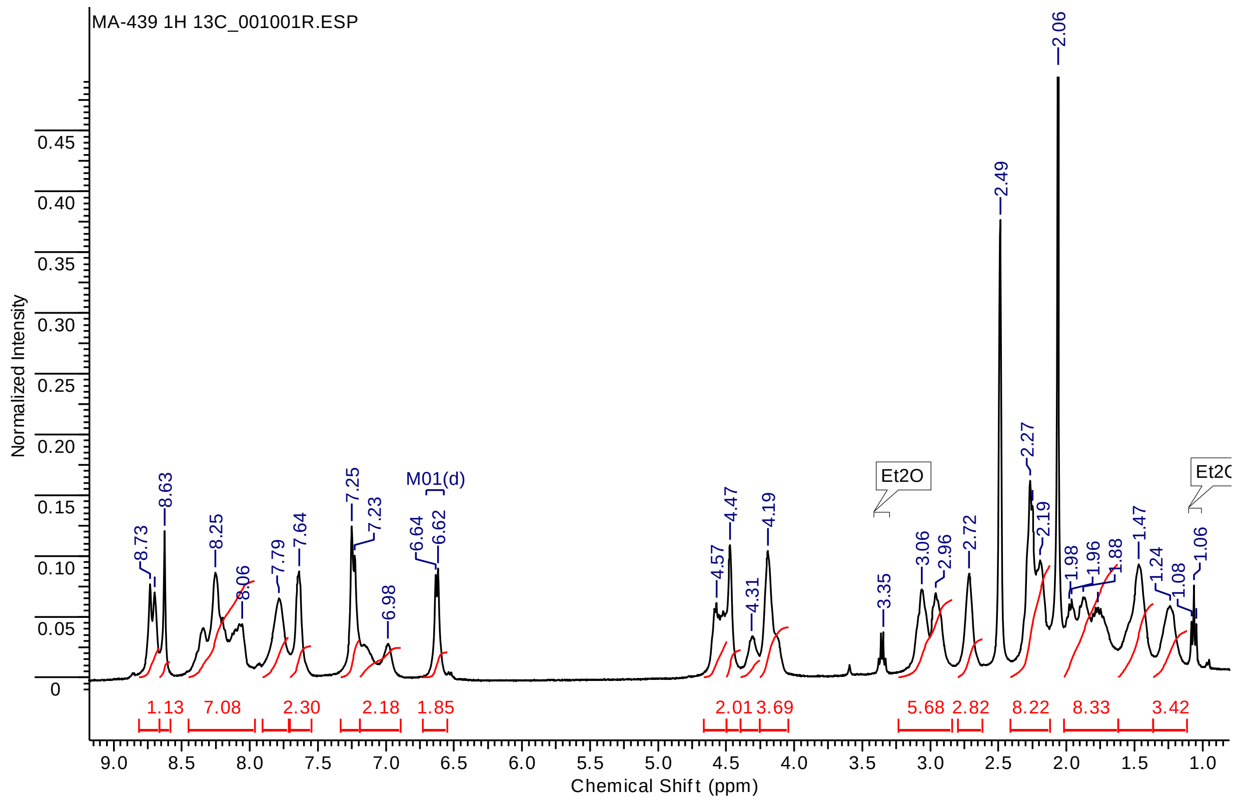

Figure S4. ^{1}H NMR spectrum (400 MHz) of **7** in DMSO-d6

HRMS (ESI)

Experiment details

High resolution mass spectra were registered by an Orbitrap Elite mass spectrometer (Thermo Scientific) with an ESI ionization source. Compounds with a concentration of 0.1–9 μg/mL (in 1% formic acid in acetonitrile) were directly infused into the ion source with a syringe pump (5 μl/min). We did not use auxiliary and sheath gases, the spray voltage was +-3.5 kV, and the capillary temperature was 275ºC. The MS spectra were registered by an Orbitrap analyzer with 480000 resolution (1 microscan, AGC target value of 1e6, max inject time 900 ms, averaged on 9 spectra, MS range 90–2000 Da, in some cases 200–4000 Da). We used DMSO and di-iso-octyl phthalate as internal calibration signals (m/z 157.03515 and 413.26623) in positive mode and dodecylsulfate (m/z 265.14790) in negative mode.

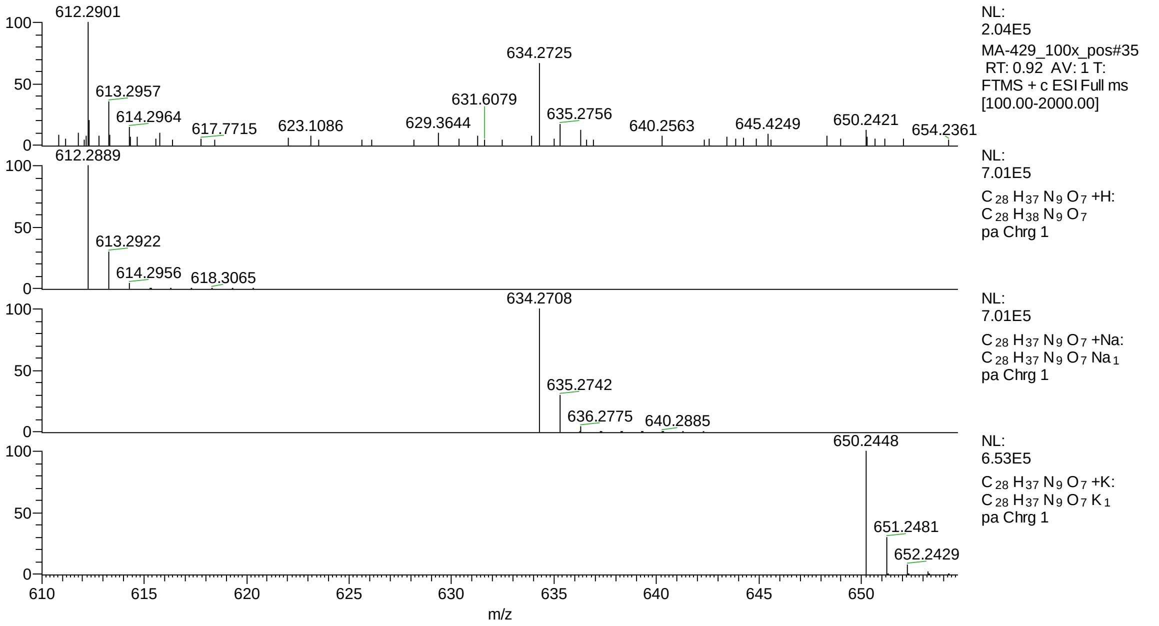

Figure S5. HRMS (ESI) spectrum of **1**

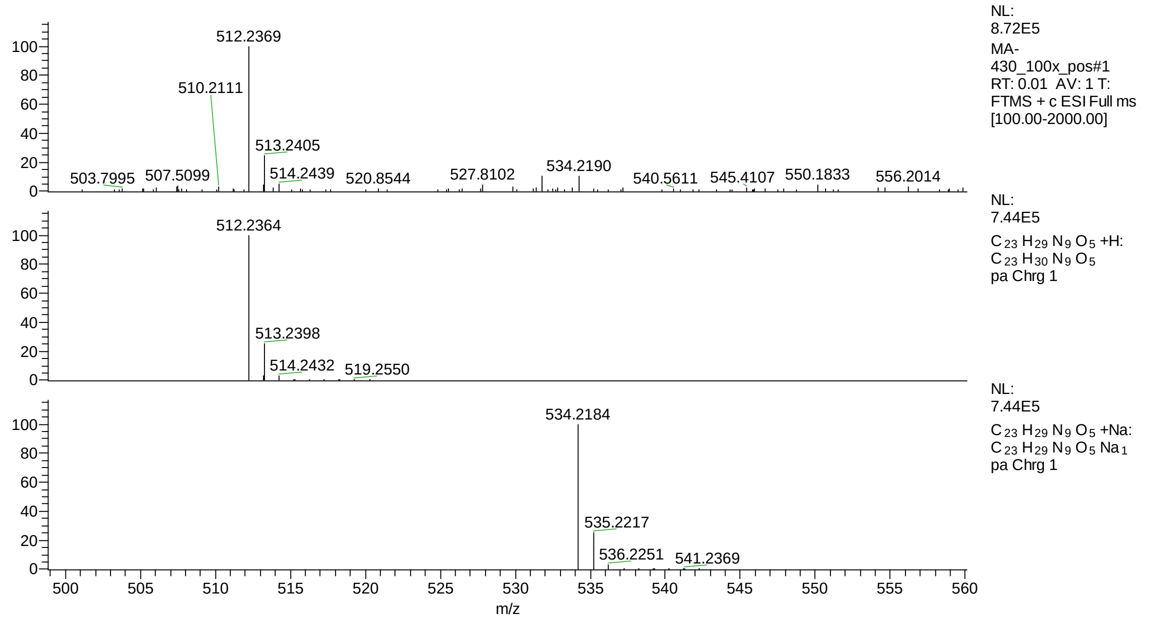

Figure S6. HRMS (ESI) spectrum of **2**

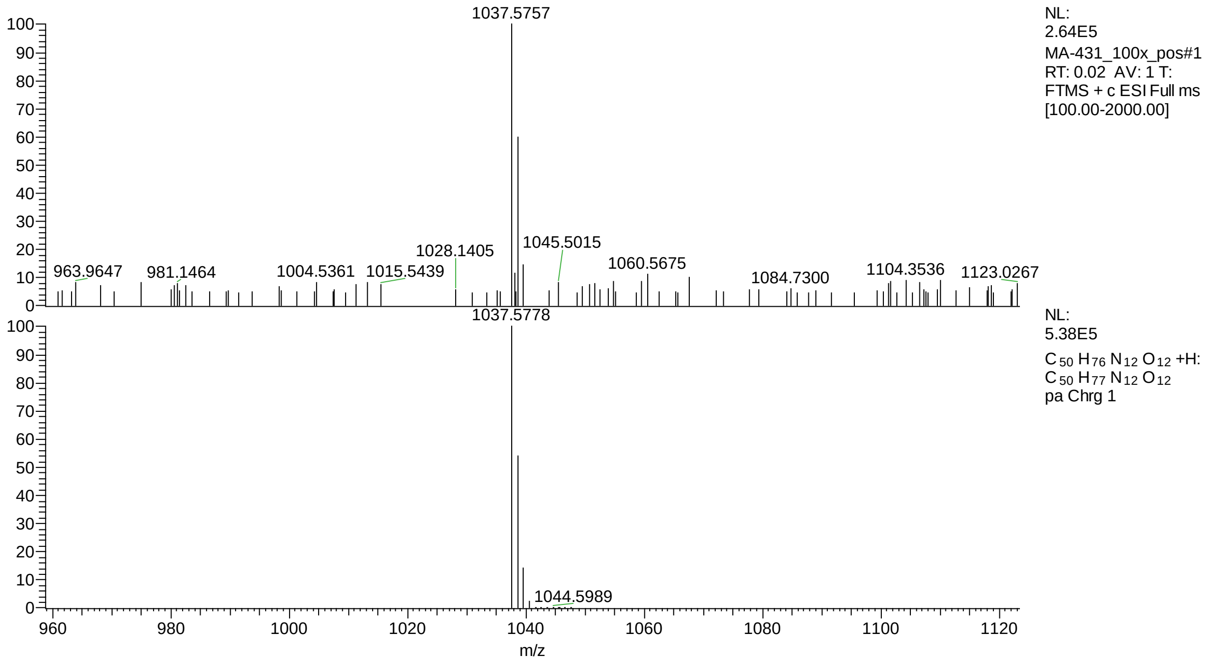

Figure S7. HRMS (ESI) spectrum of **3**

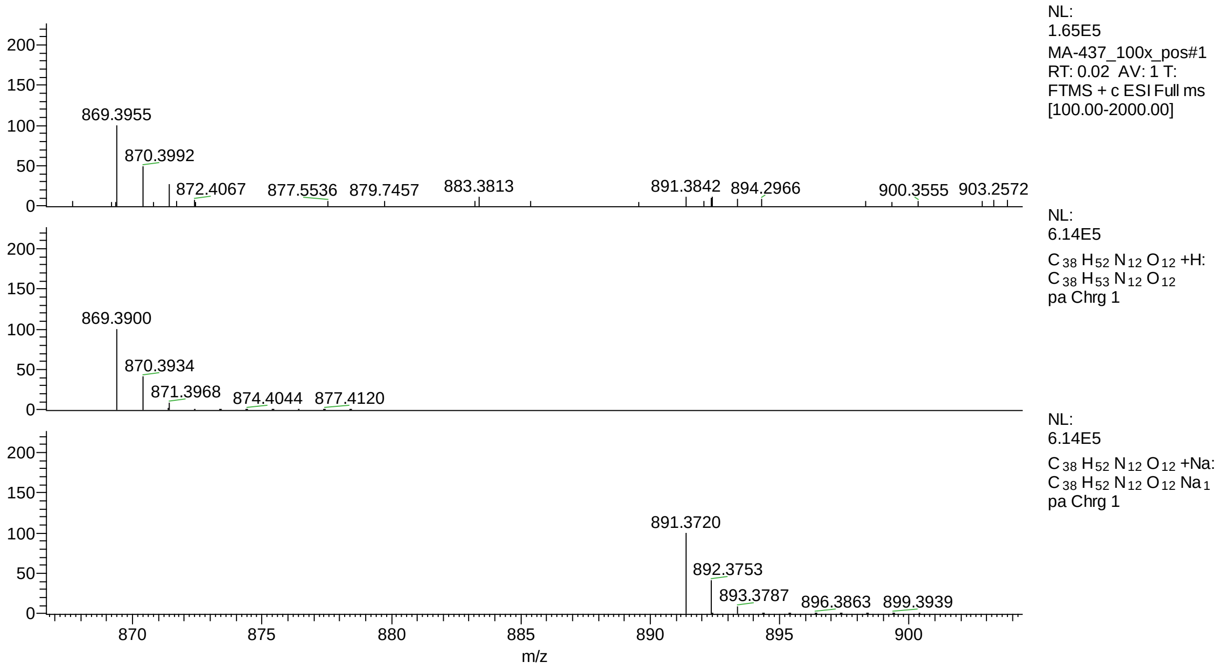

Figure S8. HRMS (ESI) spectrum of **4**

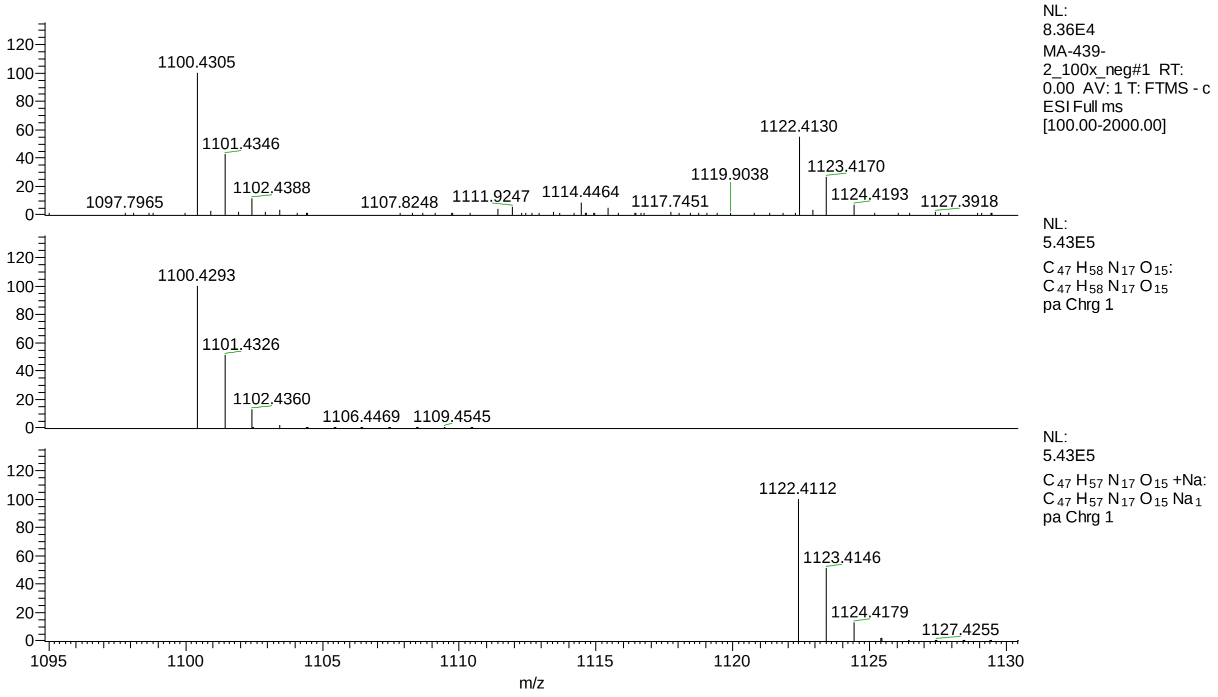

Figure S9. HRMS (ESI) spectrum of **7**

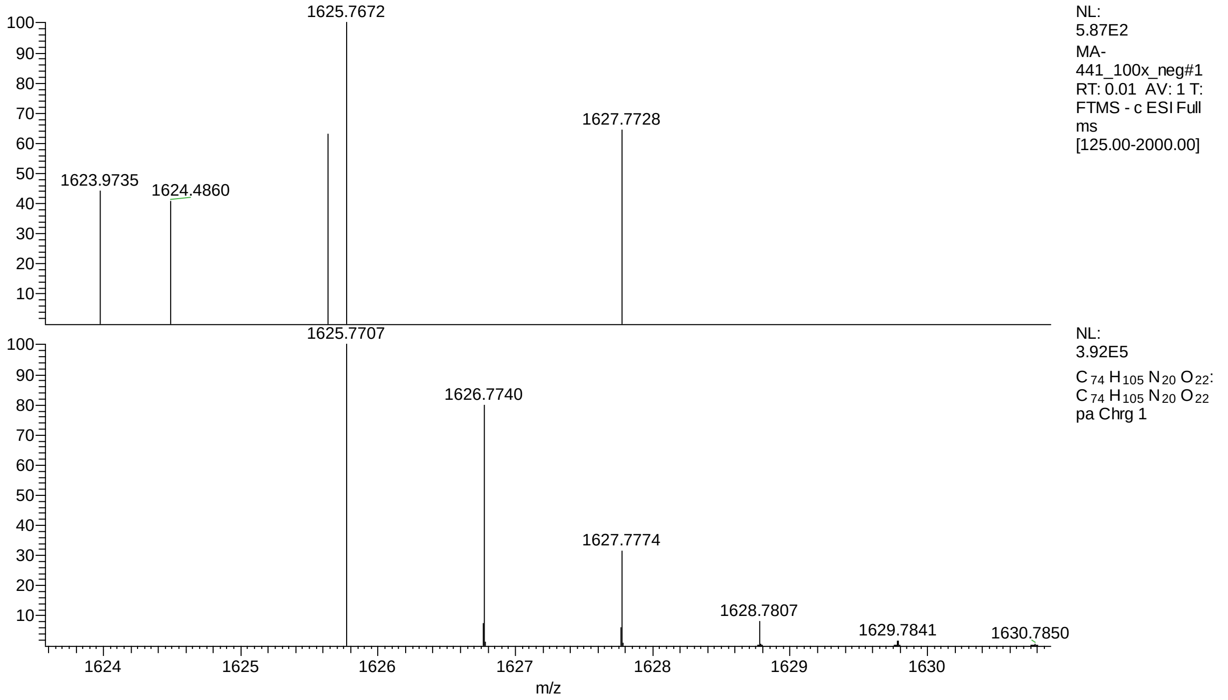

Figure S10. HRMS (ESI) spectrum of **8**

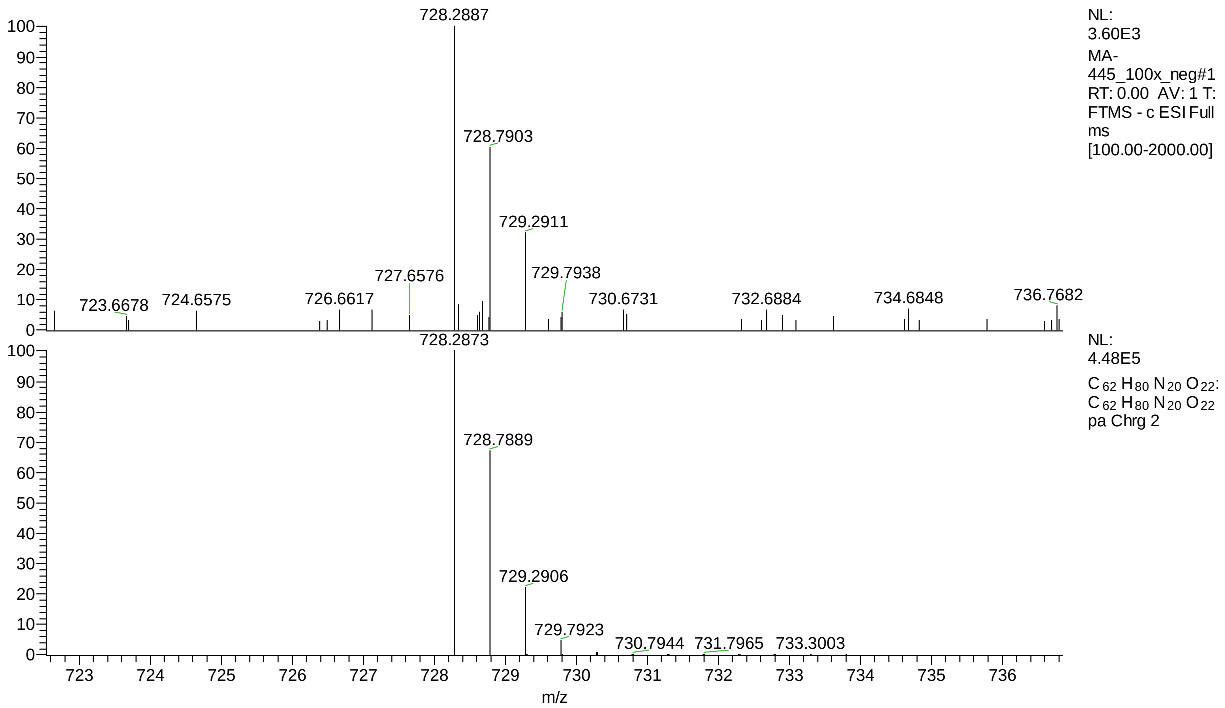

(a)

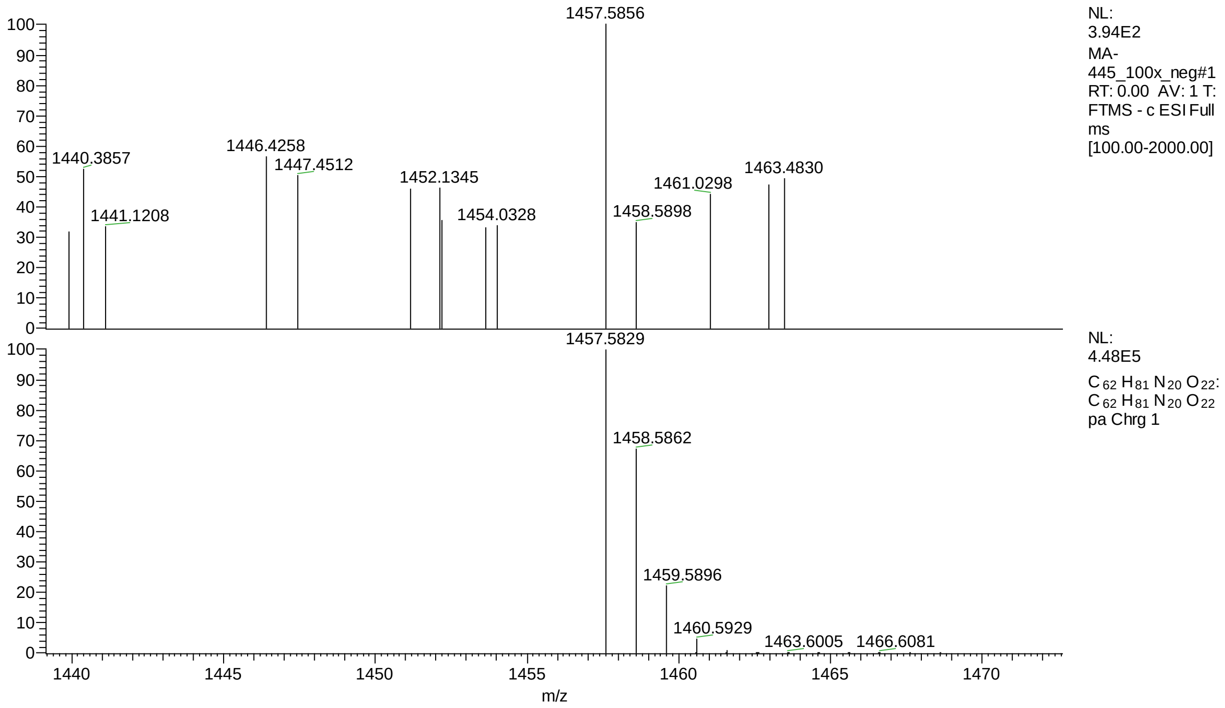

(b)

Figure S11. HRMS (ESI) spectra of **9**: double charged ion - $[M\text{-}2H]^{2-}$ (a), single charged ion $[M\text{-}H]^{-}$ (b)

HPLC-MS

Experiment details

For analysis of samples Shimadzu Prominence LC-20 system was used with Phenomenex Luna C18 column (3 µm, 90 Å, 150 x 4.6 mm) in a column oven at 40ºC and a fraction collector coupled to single quadrupole mass spectrometer Shimadzu LCMS-2020 with a dual DUIS-ESI-APCI ionization source. The mobile phases were A – 0.1% formic acid in water, D – acetonitrile. The LC parameters for analyses were a gradient flow of 1 mL/min (0–0.5 min with 5% D, 0.5–9.5 min with 5% to 90% D, 9.5–12 min with 90% D, 12–14.5 min with 90% to 5% D) with optional UV detection. The MS parameters were drying gas 15.0 L/min, nebulizing gas 1.5 L/min, DL temperature 250ºC, heat block temperature 400ºC, interface voltage -3.5 kV, and corona needle voltage -3.5 kV. Positive ions (mass range 250–2000 Da, in some cases 155–2000 Da) and negative ions (mass range 90–2000 Da) were registered simultaneously.

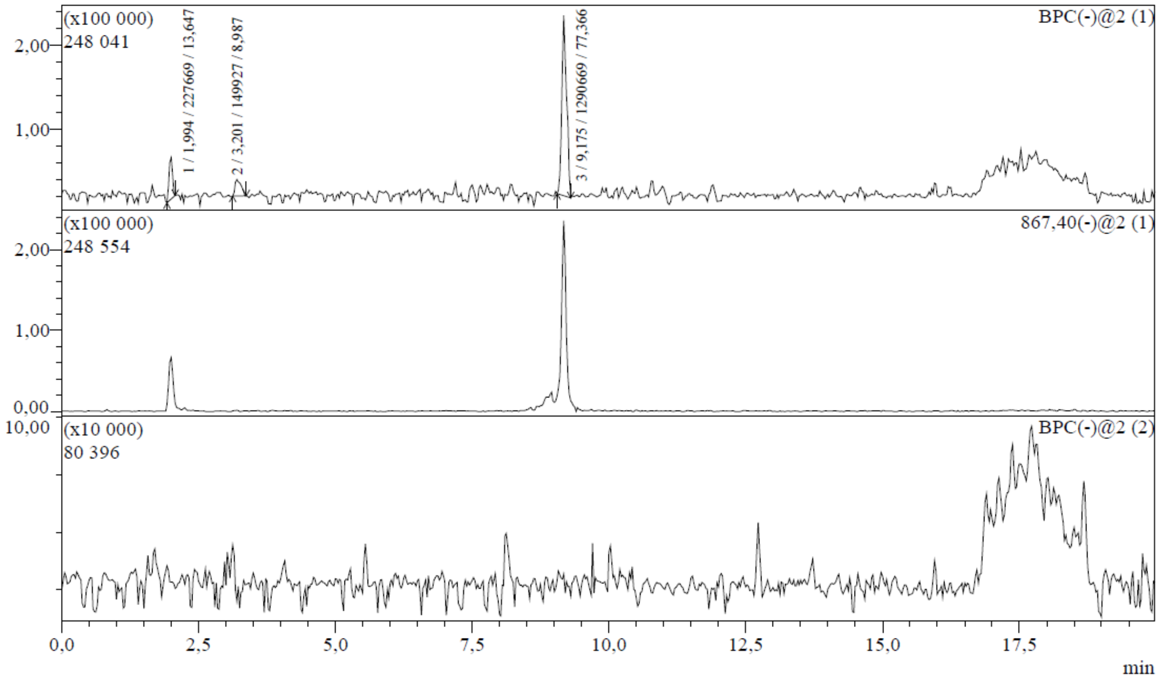

Figure S12. LC-MS chromatogram of **4 (FA-I**, top - base peak chromatogram of the sample; middle - extracted-ion chromatogram of targeted ion; bottom - base peak chromatogram of the blank sample)**.**

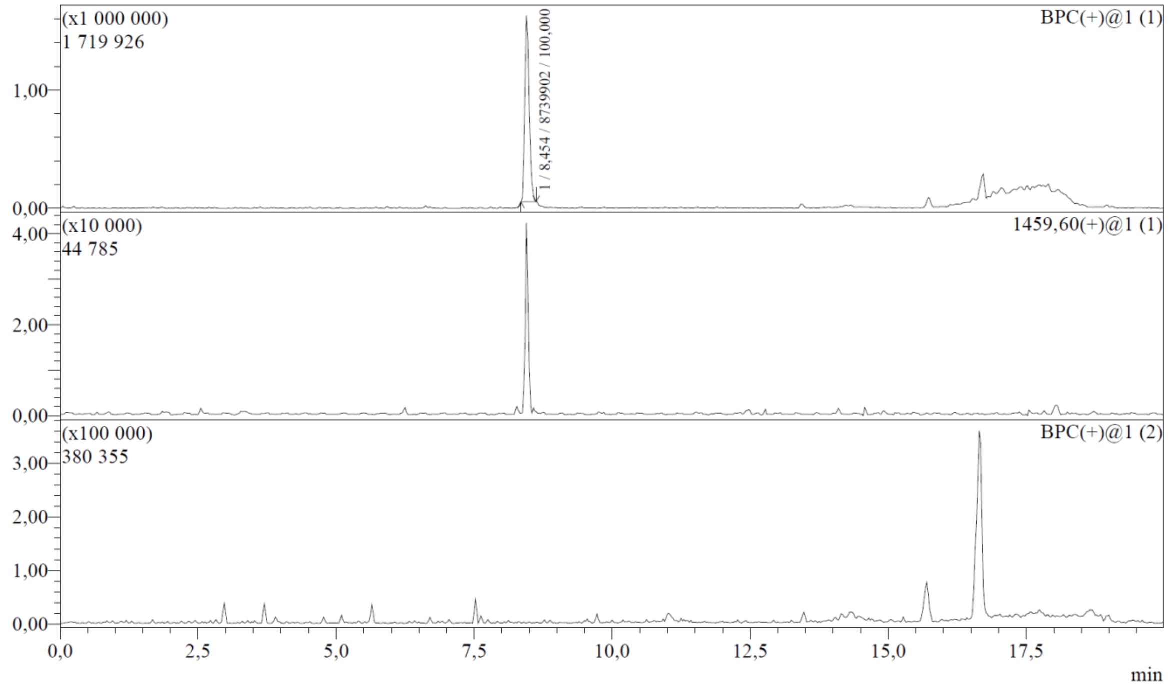

Figure S13. LC-MS chromatogram of **9 (FA-II**, top - base peak chromatogram of the sample; middle - extracted-ion chromatogram of targeted ion; bottom - base peak chromatogram of the blank sample).

Radio-TLC

Table S1. Radio-TLC chromatograms of [⁶⁸Ga]Ga-FA-I and [⁶⁸Ga]Ga-FA-II

Sample	TLC method				
	**iTLC-SG / 0.05 $H_3Citr_{aq.}$**	**iTLC-SG / 4 % $TFA_{aq.}$**	**iTLC-SG / 1 M CH_3COONH_4 (MeOH-H2O (1:1))**	**iTLC-SG / 0,08 % TFA (MeCN-saline (1:1))**	**Whatman 2 / 0,1% TFA (MeCN-saline (1:1))**
[^{68}Ga]Ga-FA-I *initial preparation in acetate buffer (pH 4.5)*	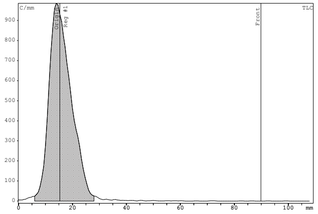	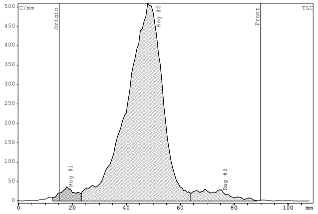	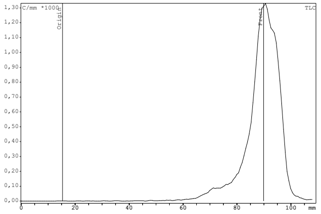	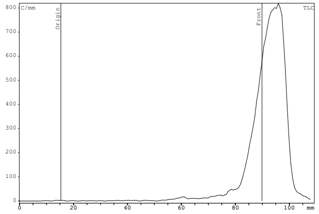	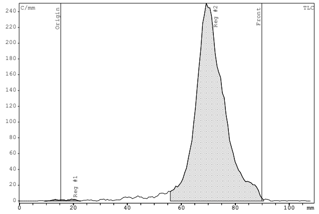
[^{68}Ga]Ga-FA-II *initial preparation in acetate buffer (pH 4.5)*	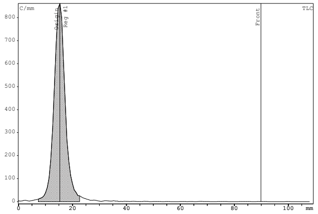	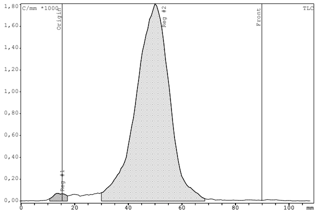	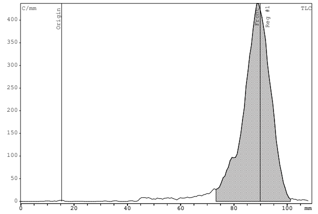	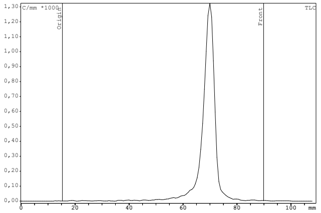	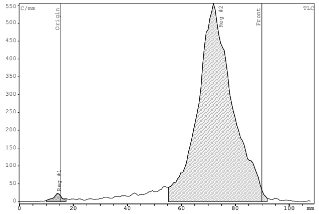
[^{68}Ga]Ga-FA-I *reformulated in saline*	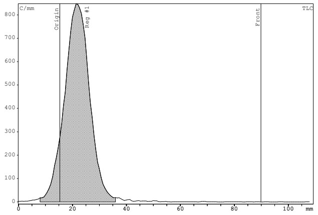	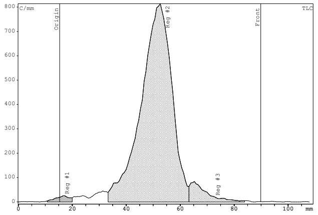	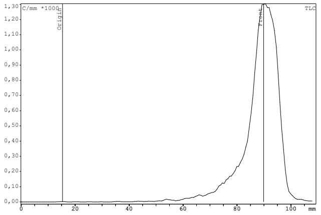	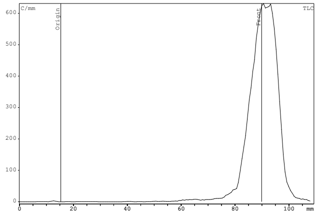	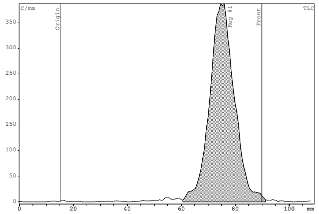
[^{68}Ga]Ga-FA-II *reformulated in saline*	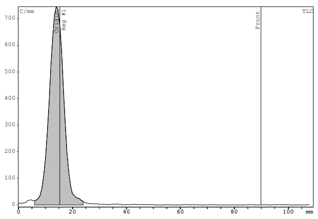	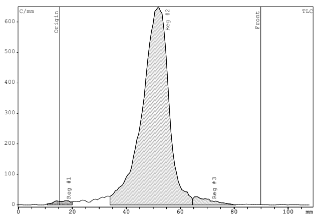	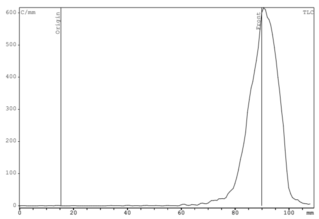	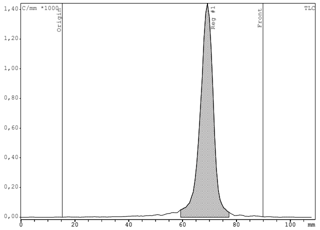	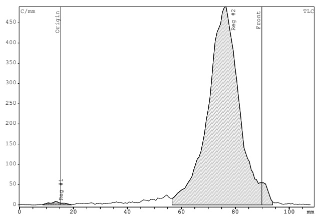
Rf (^{68}Ga-colloid)	0.0	0.0	0.0	0.0	0.0
Rf (^{68}Ga-unbound)	0.9-1.0	0.9-1.0	0.0	1.0	1.0

Table S2. Examples of radio-TLC chromatograms pre- and post-reformulation

**Sample**	**TLC method**				
	iTLC-SG / 0.05 $H_3Citr_{aq.}$	iTLC-SG / 4 % $TFA_{aq.}$	iTLC-SG / 1 M CH_3COONH_4 (MeOH-H2O (1:1))	iTLC-SG / 0,08 % TFA (MeCN-saline (1:1))	Whatman 2 / 0,1% TFA (MeCN-saline (1:1))
[68Ga]Ga-FA-II initial preparation in acetate buffer (pH 4.5)	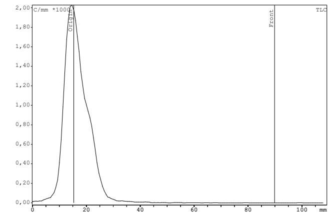	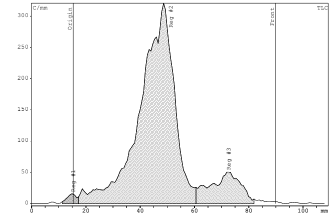	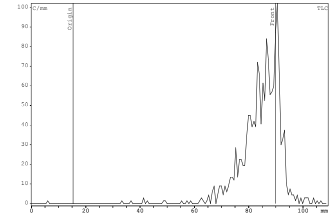	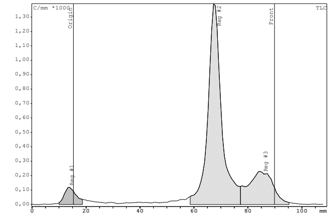	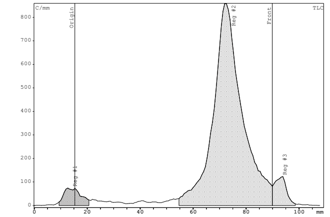
[68Ga]Ga-FA-II reformulated in saline	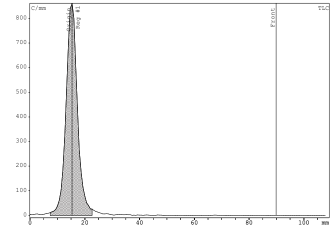	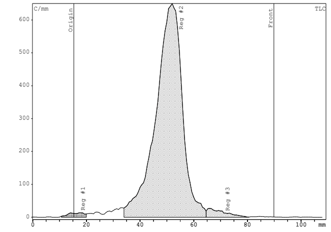	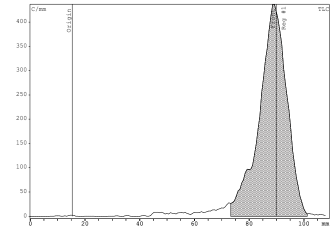	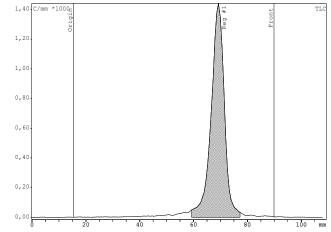	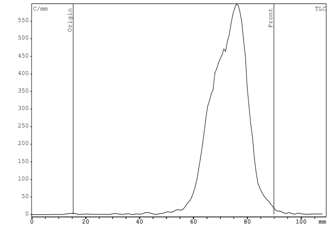

Radio-HPLC

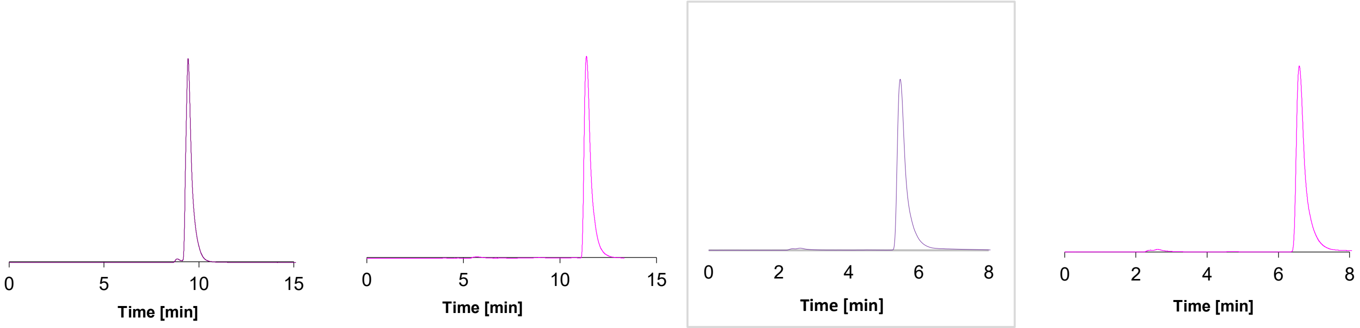

a *b* *c* *d*

Figure S14. HPLC chromatograms obtained with Method 1 for [^{68}Ga]Ga-FA-I (a) and [^{68}Ga]Ga-FA-II (b), and with Method 2 for [^{68}Ga]Ga-FA-I (c) and [^{68}Ga]Ga-FA-II (d)

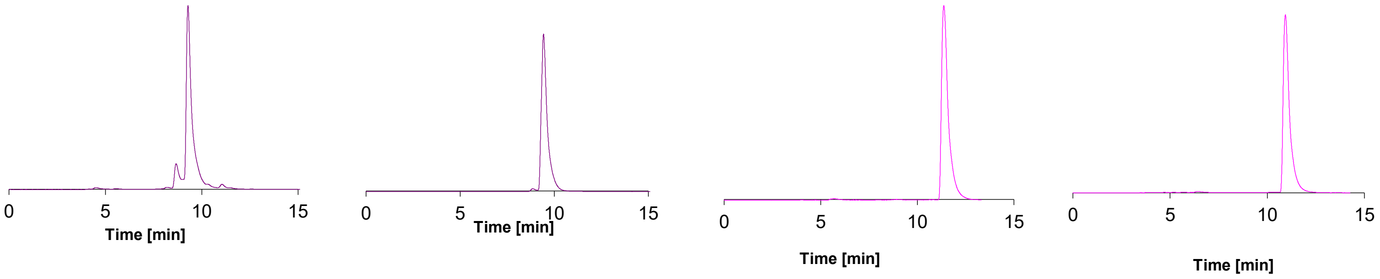

a *b* *c* *d*

Figure S15. HPLC chromatograms obtained with Method 1 for [^{68}Ga]Ga-FA-I pre- (a) and post-reformulation (b) and for [^{68}Ga]Ga-FA-II pre- (c) and post-reformulation (d)

The case of [^{68}Ga]Ga-FA-I accumulation in non-tumor neoplasm (cyst)

During the biodistribution study of [^{68}Ga]**Ga-FA-I** in immunodeficient mice with transplanted KB tumors, one of the mice autopsy (at the time point of 30 min) revealed a spontaneous neoplasm in the groin area. This neoplasm was included in the layout of organs for measurement by direct radiometry. The measurement results are presented in Table S3 (these results were not included in data presented in Tables 3, 4). Histological examination showed that this neoplasm is a cyst (Figure S16).

A small area of a large cyst surface is lined with stratified squamous epithelium, the lining is absent for the rest of it. Inflammatory infiltrates of lymphocytes and neutrophils, as well as assemblies of foamy macrophages, were found in the cyst wall. In the lumen of the cyst keratin-filled nodules, foamy macrophages and neutrophils were found. Interestingly, in addition to the high content of [^{68}Ga]**Ga-FA-I** in the blood (3.27% versus the average value of 1.37 ± 0.38%, 30 min after administration), its accumulation in the cyst was higher than that in KB tumor of the same animal (2.44% versus 1.75% ID/g).

Taking into account the data of histological examination, as well as the fact that experiments on the binding of [^{68}Ga]**Ga-FA-I** and [^{68}Ga]**Ga-FA-II** to blood leukocytes showed almost zero result (not presented), it can be assumed that the activity accumulated in the cyst is associated precisely with activated macrophages (in specific, foam cells), which is consistent with published data [8,9]. However, the uptake of the cyst is very similar to that of highly perfused organs (liver, lungs), so potentially perfusion itself could be imputable for the higher uptake in the cyst. Further research is needed to confirm the nature of this process.

Table S3. Biodistribution of [^{68}Ga]**Ga-FA-I** in KB-tumor bearing BALB/c nude mouse with spontaneous neoplasm 30 min after injection

Tissue/ Organ	ID/g, %
blood	3.27
lungs	2.83
heart	1.51
stomach	0.67
spleen	0.89
liver	2.48
kidneys	14.68
bladder	1.19
intestines	1.07
brain	0.18
muscle	0.37
tumor (KB)	1.75
neoplasm (cyst)	2.44

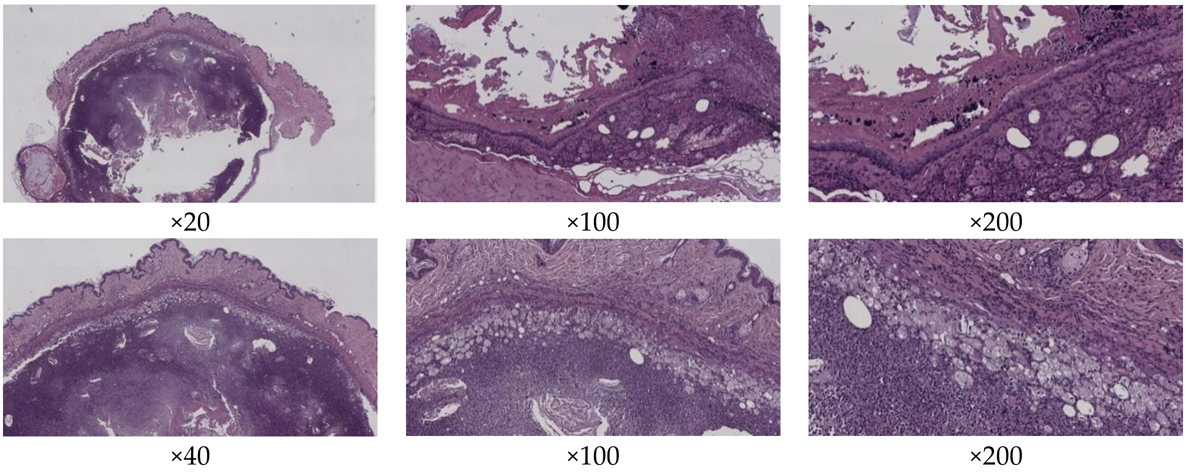

Figure S16. Micrographs of spontaneous neoplasm from the groin of the mice at various magnifications. The histological picture corresponds to epidermal cyst of the skin with signs of suppuration of the wall.

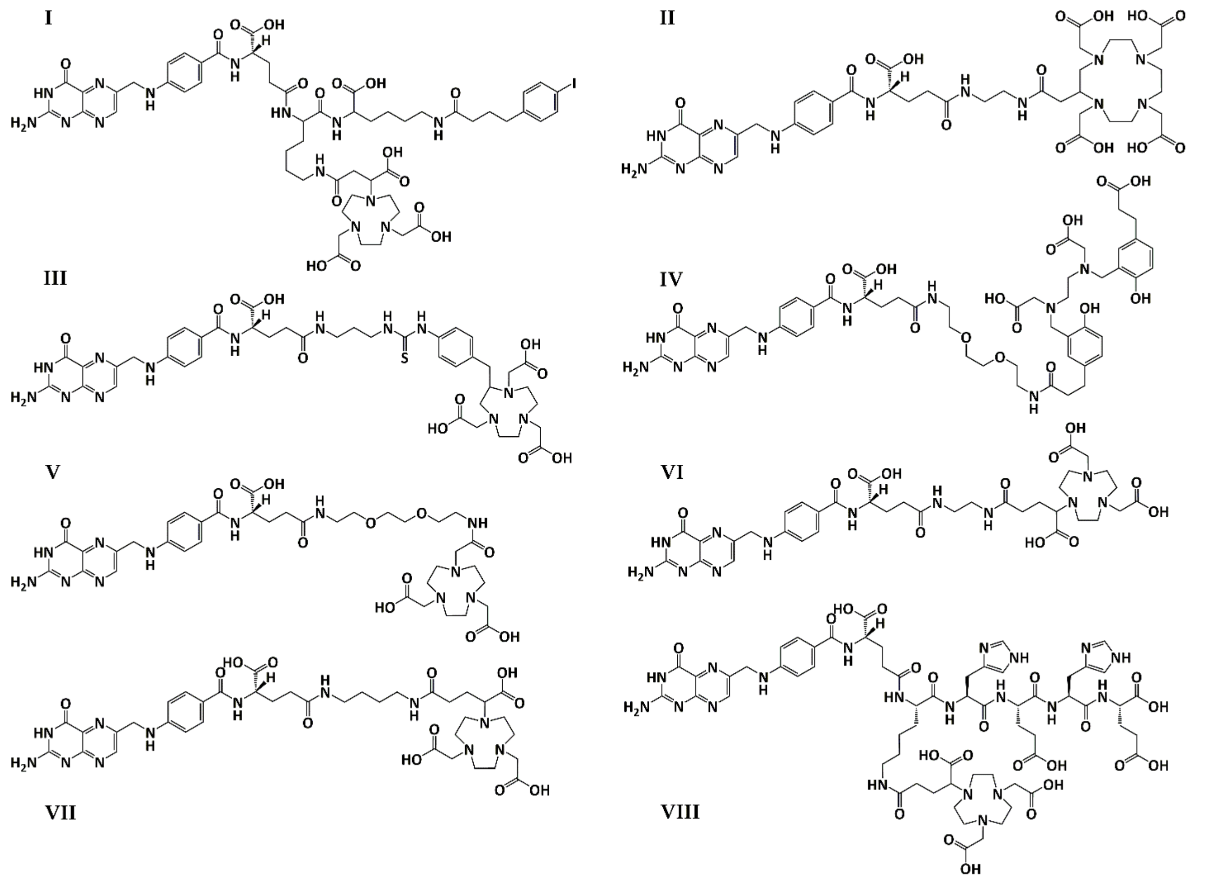

Figure S17. Chemical structure of [^{68}Ga]**Ga-FA-I** (**VII**) and [^{68}Ga]**Ga-FA-II** (**VIII**) in comparison with other published folate-based conjugates tested with radiogallium (for references see **Tables 5, 6**).
